# An overview of DNA barcoding of biodiversity in South Africa

**DOI:** 10.1371/journal.pone.0345173

**Published:** 2026-04-21

**Authors:** Mahlatse M. Kgatla, Cassandra Barker, Janine R. Baxter, Aletta E. Bester-van der Merwe, Mamohale Chaisi, Albert Chakona, Michael I. Cherry, Savel R. Daniels, Louis H. Du Preez, Charles R. Haddad, Peter G. Hawkes, Chris Ho, Thierry B. Hoareau, Adriaana Jacobs, Karin Jacobs, Charlene Janion-Scheepers, Bettine Jansen van Vuuren, Ronny M. Kabongo, Thembile T. Khoza, Nozipho L. Khumalo, Tendekai Mahlanza, Lindiwe Makapela, Buyisile G. Makhubo, Gavin W. Maneveldt, Kagiso Mashego, Gwynneth Matcher, Conrad A. Matthee, Mudzuli Mavhunga, John M. Midgley, Musa Mlambo, Daniela M. Monsanto, Rungula Mthombeni, Shane L. Murray, Samantha Mynhardt, Brielle-Jennifer Nang-Mba, Mduduzi Ndlovu, Shilpa P. Parbhu, Veronica Phetla, Metlholo Phukuntsi, Tristan R. Pitcher, Toufiek Samaai, Carol A. Simon, Kerry Sink, Catherine L. Sole, Genevieve L. Theron, Barbara van Asch, Michelle van der Bank, Clarke J.M. van Steenderen, Martin H. Villet, Cobus M. Visagie, Kirstin A. Williams, Sandi Willows-Munro, Mamadi T. Sethusa, Jessica M. Da Silva, Monica Mwale

**Affiliations:** 1 South African National Biodiversity Institute, Pretoria, Gauteng, South Africa; 2 Department of Botany and Zoology, Stellenbosch University, Stellenbosch, Western Cape, South Africa; 3 Department of Natural Sciences, KwaZulu-Natal Museum, Pietermaritzburg, KwaZulu-Natal, South Africa; 4 Department of Genetics, Stellenbosch University, Stellenbosch, Western Cape, South Africa; 5 Department of Veterinary Tropical Diseases, University of Pretoria, Pretoria, Gauteng, South Africa; 6 South African Institute for Aquatic Biodiversity, Makhanda, Eastern Cape, South Africa; 7 Department of Ichthyology and Fisheries Science, Rhodes University, Makhanda, Eastern Cape, South Africa; 8 Unit for Environmental Sciences and Management, North-West University, Potchefstroom, North-West, South Africa; 9 Department of Zoology & Entomology, University of the Free State, Bloemfontein, Free State, South Africa; 10 Afribugs CC, Pretoria, Gauteng, South Africa; 11 Department of Biological Science, University of Venda, Thohoyandou, Limpopo, South Africa; 12 Centre for Biodiversity Genomics, University of Guelph, Guelph, Canada; 13 Department of Biochemistry, Genetics and Microbiology, University of Pretoria, Pretoria, South Africa; 14 Reneco International Wildlife Consultants LTD, Abu Dhabi, United Arab Emirates; 15 Department of Terrestrial Invertebrates, National Museum, Bloemfontein, Free State, South Africa; 16 Department of Botany & Plant Biotechnology, University of Johannesburg, Johannesburg, Gauteng, South Africa; 17 Department of Microbiology, Stellenbosch University, Stellenbosch, Western Cape, South Africa; 18 Department of Biological Sciences, University of Cape Town, Cape Town, Western Cape, South Africa; 19 Iziko Museums of South Africa, Cape Town, Western Cape, South Africa; 20 Centre for Ecological Genomics and Wildlife Conservation, University of Johannesburg, Johannesburg, Gauteng, South Africa; 21 School of Life Sciences, University of KwaZulu-Natal, Durban, KwaZulu-Natal, South Africa; 22 Department of Biodiversity and Conservation Biology, University of the Western Cape, Bellvile, Western Cape, South Africa; 23 Department of Biochemistry and Microbiology, Rhodes University, Makhanda, Eastern Cape, South Africa; 24 Department of Zoology and Entomology, Rhodes University, Makhanda, Eastern Cape, South Africa; 25 Department of Freshwater Invertebrates, Albany Museum, Makhanda, Eastern Cape, South Africa; 26 DIPLOMICS, Cape Town, Western Cape, South Africa; 27 School of Biology and Environmental Sciences, University of Mpumalanga, Mbombela, Mpumalanga, South Africa; 28 South African Environmental Observation Network, Cape Town, Western Cape, South Africa; 29 Department of Forestry, Fisheries and Environment, Oceans and Coasts Research, Cape Town, Western Cape, South Africa; 30 Institute for Coastal and Marine Research, Nelson Mandela University, Gqeberha, Eastern Cape, South Africa; 31 Department of Zoology and Entomology, University of Pretoria, Pretoria, Gauteng, South Africa; 32 Department of Conservation Ecology and Entomology, Stellenbosch University, Stellenbosch, Western Cape, South Africa; 33 Department of Biochemistry Genetics and Microbiology, Faculty of Natural and Agricultural Sciences, Forestry and Agricultural Biotechnology Institute (FABI), University of Pretoria, Pretoria, South Africa; 34 School of Life Sciences, University of KwaZulu-Natal, Pietermaritzburg, KwaZulu-Natal, South Africa; University of Padova: Universita degli Studi di Padova, ITALY

## Abstract

The global decline in biodiversity, driven by habitat loss, overexploitation, climate change, biological invasions, and illegal trade, poses significant challenges for conservation management. Although many South African ecosystems and species are under threat, effective conservation efforts are hindered by incomplete foundational biodiversity data and assessments, caused by taxonomic gaps and unverified distributions. DNA barcoding has emerged as an invaluable tool for species identification and classification of biodiversity. While substantial barcoding progress has been made, for many taxa, others remain underrepresented in sequence databases. This study evaluates the status and progress of DNA barcoding in South Africa through a gap analysis, comparing verified species checklists with barcoded sequences from the Barcode of Life Database (BOLD) and GenBank to assess taxonomic and geographic representation. A literature review (2003–2023) highlights applications across terrestrial, freshwater, and marine habitats. Of the 931,476 South African species barcode records, 52% were publicly available. Although the insects dominated with the highest number of records and BINs, reptiles had the highest taxonomic representation. Plants and fungi were underrepresented (16.1% and 2.8%, respectively). Regionally, Mpumalanga and Limpopo provinces showed the highest BIN counts, while North-West and Free State provinces had the lowest. The majority of barcode records were for mtDNA genes such as cytochrome c oxidase subunit I (*COI*) and were contributed by both local and international institutions. Discrepancies between GenBank records and those mined by BOLD indicated that many GenBank sequences for South Africa have poor quality metadata, including geographic sampling locality information. While significant progress has been made across taxa, further efforts are needed to expand species and geographic coverage, enhance sequence quality, improve species metadata, and resolve inconsistencies in BIN assignments, particularly for underrepresented groups such as plants and fungi. These advances would strengthen biodiversity assessments and support conservation efforts in South Africa.

## Introduction

### DNA barcoding for monitoring biodiversity

South Africa is one of the most biologically diverse countries in the world, with ecosystem types ranging from alpine to marine and freshwater to desert [1]. Moreover, the country harbours three of the 35 globally recognised biodiversity hotspots namely the Cape Floristic Region, the Succulent Karoo, and the Maputaland-Pondoland-Albany corridor [2,3]. Unfortunately, South Africa has not been spared from biodiversity loss: the 2018 National Biodiversity Assessment [1] indicated that almost half of the 1,021 assessed ecosystems are threatened, and that 31% of all ecosystem types fall outside protected areas. The same report categorised 14% of the 2,856 known plant species and 12% of animals (approximately 350 species) as threatened according to the national assessment of status. However, many taxonomic groups remain unassessed or data deficient, either due to incomplete descriptions or misclassification, or because of insufficient information regarding distribution, population size, and/or threats [4,5]. Consequently, South Africa’s biodiversity will remain underestimated unless efforts are made to correctly identify and comprehensively curate taxa [6].

Documenting global biodiversity is resource-intensive and has progressed slowly under traditional morphology-based taxonomy, with probably lesser than 14% of species described after more than two centuries [7–9]. Limitations such as accessibility, time, and taxonomic expertise led to the establishment of the Global Taxonomy Initiative in 1998 and a shift toward a more integrative taxonomic identification by incorporating DNA sequences [6,7,10,11]. DNA barcoding was proposed over just 20 years ago as a tool to aid in species resolution and delineation [12]. Through the International Barcode of Life project (iBOL), the Barcode of Life Data System (BOLD) was established and has enabled the development of global reference libraries that support biodiversity assessments and species inventories [13]. This practice, informed primarily by the phylogenetic species concept [14], uses the Barcode Index Number system (BINs) to cluster animal *COI* sequences into operational taxonomic units that generally correspond to biological species, which typically aligns closely with biological species [13,15,16].

DNA barcoding employs standardized genetic markers, including the mitochondrial *cytochrome c oxidase I* (*COI*) Folmer region in animals [17,18], plastid *maturase K* (*matK*) and *ribulose 1,5-bisphosphate carboxylase* (*rbcLa*) in plants [19], and nuclear ribosomal internal transcribed spacer (ITS) in fungi [20], which generally have higher interspecific and lower intraspecific variation [12]. However, there is lower discriminatory power in some taxon groups such as plants and fungi as some taxa require additional secondary markers for more accurate barcode species delimitation [21,22]. Despite early controversy regarding the barcode gap analysis, use of single DNA marker for identifications and error rates associated with incomplete reference databases [23], DNA barcoding has still proven effective for species identification and delimitation for informing species-level systematics. Its application has expanded from traditional taxonomy to biodiversity assessment, conservation, invasive species surveillance, wildlife forensics, and more recently, high-throughput approaches such as metabarcoding for community-level analyses and monitoring [24–27]. One notable taxonomic application has been the use of DNA barcodes generated from type specimens (and other museum vouchers) to accurately verify or apply names to contemporary specimens, rather than relying solely on morpho-anatomical similarity [28]. Therefore, the effective utilisation of DNA barcoding rests on reliable reference libraries of species that contain verified, high-quality DNA sequence data obtained from taxonomically certain voucher specimens of known taxa [11,15,28].

### DNA barcoding campaigns in South Africa

In Africa, relatively little biodiversity has been catalogued compared to other continents [1,29], yet the pressures threatening its existence are immense [10]. Consequently, there is great scope for DNA barcoding, and there have been some initiatives to promote barcoding in the continent, such as iBOL grants and initiatives in South Africa [29,30]. Through iBOL, South Africa established the South African Node of the International Barcode of Life (SABOL) in 2010, which aims to promote DNA barcoding, implement international priorities of iBOL working groups, encourage collaboration within the biodiversity conservation community, identify national priorities for barcoding research, and bridge gaps in the reference library on BOLD while maximising the use of available resources and capacity (https://fbip.co.za/about/sanbi-gbif/). Since 2013, DNA barcoding in South Africa has also been supported by the Foundational Biodiversity Information Programme (FBIP) [31,32], which aims to fund the generation, mobilisation and integration of priority foundational biodiversity data, including DNA barcoding. The FBIP (https://fbip.co.za/) has prioritised previously neglected taxa (e.g., fish, marine invertebrates, macroalgae, freshwater organisms, soil microorganisms) and understudied geographic regions in South Africa. A 2016 review on the progress of DNA barcoding of South Africa’s fauna, with a specific focus on BOLD, indicated that just under 48,000 barcodes had been made publicly available by 2014, primarily from insects [33]. These barcodes represented only 2.3% of all globally known animal species and just over 1% of the insects. Among the vertebrates, taxonomic coverage was biased towards fish, birds, and mammals [33]. Geographic bias was also evident, with most records coming from provinces with greater scientific and technical resources rather than being proportional to the level of biodiversity in those areas. Several suggestions were made to increase South African barcode representation on BOLD and address the taxon and sampling gaps [33]. These gaps could potentially be mitigated by utilising other nucleotide sequence databases, such as the European Molecular Biology Laboratory (EMBL) database maintained by the European Bioinformatics Institute (EBI), the DNA Data Bank of Japan (DDBJ) maintained by Japan’s National Institute of Genetics, and the GenBank database maintained by the U.S. National Center for Biotechnology Information [34–36]. The EMBL-EBI and DDBJ serve as region-centric databases, while GenBank serves as a globally comprehensive sequence database. These databases now have data-sharing agreements.

This review seeks to evaluate the current status of barcoding in South Africa as of October 2023, with a particular emphasis on assessing the country’s contributions to the DNA barcoding species reference library. The primary objectives were to: (1) conduct a comprehensive analysis of the progress made on BOLD over the past decade in addressing taxonomic and geographic coverage gaps of DNA barcoding; (2) quantitatively assess the contribution of complementary non barcode standard sequence data of South African species in other molecular databases such GenBank; and (3) examine the key challenges and opportunities associated with DNA barcoding applications in South Africa, including barriers to adoption and technological limitations. These findings will inform the evidence-based recommendations for strategic prioritisation in the generation of biodiversity data through the South African Barcode of Life (SABOL) node.

## Materials and methods

### Verification of checklists

A national plant checklist (S1 Table) was downloaded from the South African National Biodiversity Institute (SANBI) checklist of southern African plant names and floristic details (Downloaded 2 November 2023; https://posa.sanbi.org/) [37]. Fungi checklists were sourced from various databases, including BOLD Systems [13], GenBank, the Global Biodiversity Information Facility (GBIF; downloaded 07 November 2023; https://doi.org/10.15468/dl.7mmzrq), the Index Fungorum database (IF; downloaded 16 January 2024; https://www.indexfungorum.org), and a published fungus checklist [38]. All searches for fungal checklists on these repositories were conducted using the search terms “fungi” and “South Africa”. Due to the methodological approach employed, records lacking information on country of origin or taxonomic classification at kingdom level would have been missed. After data acquisition, each list was sorted alphabetically, and species names were cross-compared to identify and remove exact duplicates. Author citations were then removed, and species names were concatenated into a single standardised string comprising the genus and specific epithet, including subspecies, varieties, and unique numerical identifiers, to enable further duplicate detection. A fuzzy matching approach was subsequently applied to identify potential typographical and spelling inconsistencies, which were verified manually. In cases of conflict, nomenclature from authoritative sources, including Index Fungorum and published checklists, was adopted. This process resulted in the final fungal checklist (S2 Table) used in the study. Checklists of South African animal taxa were sourced from taxon experts in collaboration with SANBI. These checklists are compiled under the guidance of a national checklist committee and are verified and cross-referenced before publication. In addition, a checklist of known alien (introduced) species (S3 Table) was sourced and compiled from a report produced by the Centre for Invasion Biology (CIB) at Stellenbosch University and SANBI [39,40], BOLD, and iNaturalist [41]. The checklist of alien taxa was used to avoid counting aliens as native species. All checklists were verified, checked for duplicates, standardised to species level (i.e., taxa below species level were collapsed for the purpose of this study), and compiled into a final dataset using MS Excel (Microsoft 365). Uncertain species identifications with “cf.”, “aff.” or “nov.” qualifiers were considered as unique species names as they may represent undescribed taxa. Checklist records where only a species name was given instead of a full taxonomic classification (i.e., kingdom, phylum, class, order, family, genus, species) were automatically completed using the GBIF taxonomic backbone through the network’s Species API v1. Over 600 inconclusive records from Species API were individually checked to verify the validity of the species name.

### Evaluating progress of addressing gaps in barcode records on BOLD

DNA barcode records of South African species published by October 2023 were mined and downloaded from BOLD by using the search term “South Africa” in the geography field. However, we note that this may have excluded records with erroneous geographic data. Therefore, a summary of private DNA sequence records and all other national records was requested from BOLD to quantify the extent of inaccessible barcoding data. The resulting records were further filtered and cleaned by removing fields (columns) considered irrelevant to the study. The following data fields were retained and used for downstream analyses of the BOLD dataset: processed, year, sampleid, institution_storing, bin_uri, kingdom, phylum_name, class_name, Order_name, family_name, genus_name, species_name, subspecies_name, lat (latitude), lon (longitude), country, province_state, markercode, genbank_accession. The filtered dataset was then divided into taxonomic groups following the classification system used in the South African “National Strategy for Zoological Taxonomy” report [6]. Plants and fungi were not subdivided further due to the lack of BINs for these groups. A similar approach to that of the checklist processing was applied to the data from BOLD, for uncertain but unique species identifications. Additionally, taxonomic classification was collapsed to species level for records identified below species level and to collapse synonyms. This standardised the data to easily perform comparisons between the checklist and the data from the BOLD dataset.

Several metrics were quantified for each taxonomic group: (i) the total number of records on BOLD; (ii) the number of distinct records (unique voucher specimens) with iBOL-compliant DNA barcode sequence data (DNA sequences that meet the standards, policies, and protocols established for DNA barcodes as outlined by iBOL) for each of the barcoding gene regions; and (iii) the number of BINs on BOLD only for *COI* barcode region [17,19,20]. To identify gaps in the barcode records, the checklist was cross-referenced against BOLD for each taxon group to determine the percentage of known species with records on the database. To assess the accuracy of taxonomic assignments, BIN discordances were assessed by considering BIN splits (species name with more than one BIN on BOLD) and BIN merges (more than one species name included in a BIN). BIN splits and merges highlight areas of taxonomic interest, as discordant BIN assignments may indicate inconclusive species delineation, hidden diversity or misidentifications [15]. Furthermore, we estimated the number of specimen records without BINs or barcodes to identify potential datasets that might be available for processing (i.e., backlogs of data that require capacity to deposit barcodes on BOLD) or to highlight issues with the barcoding process.

### Geographic distribution and contribution of *COI* BOLD data

To investigate the geographical representation of South African BOLD barcode records with a BIN number, the locality data were verified and grouped according to provincial boundaries. Non-BIN barcode data were excluded from this analysis as these *COI* records still require verification with phylogenetic assessment for species identifications, which excluded large number of records particularly plants and fungi (which contain non-*COI* records). Records lacking GPS coordinates, but containing a province_state data field, were included by generating a centroid coordinate for the province in Quantum Geographic Information System (QGIS.org version 3.28.7). Each province’s contribution was then quantified for each taxon group. The countries, regions, and institutions contributing DNA barcodes of South African voucher specimens (verified by GPS coordinates) were recorded based on information obtained from the [institution_storing] data field to identify the levels of (local, regional, and international) contributions towards barcoding efforts of South Africa’s biodiversity. Additionally, the extent of institutional representation and contributions was highlighted.

To further quantify the contribution of DNA barcoding of South African taxa, an investigation of peer-reviewed scientific publications was undertaken, focusing specifically on publications between 2003 and 2023, after the publication of Hebert et al (2003) [17]. Only publications that generated DNA barcodes recognised by iBOL or used at least one barcode marker to improve or undertake identification were considered. The collation of publications was achieved by conducting literature searches on Science Direct, Web of Science, Google Scholar, and PubMed, using the search terms “South Africa” and “[molecular]” (e.g., DNA sequencing, or genetic diversity, or molecular analyses, or phylogenetics, or reference library, or species delimitation, *cytochrome oxidase subunit I, COI*). The compiled information was exported to MS Excel to verify accuracy and remove duplicates (S4 Table). The publications were separated into taxonomic groups and then divided according to two distinct sets of criteria over four time periods (at five-year intervals): (1) Publications that had generated barcodes (i.e., publications that produced barcode data) or not (total publications, including those without barcode data); and (2) publications on environmental studies (Terrestrial, Marine, or Freshwater).

### Evaluating the current status of barcode records on GenBank

GenBank was searched using advanced searches, applying the terms “South Africa” and “[taxon group]” (e.g., reptile, or mammal, or plant, or fungus). The resulting records were downloaded as text files and subsequently uploaded to MS Excel for further processing (S1 File). However, some GenBank datasets were too large to process locally in MS Excel, requiring the use of Row Zero (https://rowzero.io) as a more efficient cloud-based solution. The entire complement of South African sequences downloaded directly from GenBank was also compared to the BOLD records to assess the discrepancy between records mined from GenBank by BOLD (for approved barcode gene regions) and total GenBank records. The total number of BOLD records annotated with the tag “Mined from GenBank, NCBI” in the “institution_storing” field was compared to the total number of records retrieved directly from GenBank, filtered by standard barcode markers (*COI* for animals, *matK* and *rbcLa* for plants, and ITS for fungi).

## Results

### Analyses of South African checklists versus the BOLD dataset

The compiled checklists have a total of 92,287 species native to South Africa, with animals having the highest representation with 58,182 species (63%), followed by plants with 21,524 species (23%) and fungi with 12,581 species (14%). A total of 931,476 South African specimen records were available on BOLD (Table 1), representing ~7% of the total global records (~13 million) available as of October 2023. Of these, 489,857 records (52%) are publicly accessible, while 441,619 records (48%) remain privately held (Table 1, S2 Fig). The public records represent 9,064 species, 10% of the estimated number of species on the compiled South African checklist. Only publicly accessible records were analysed, as privately held records could not be accessed. From the public records, 388,608 were assigned barcode index numbers (34,660 unique BINs), with 131,249 being unassigned. Assuming that each BIN represents a distinct species lineage (Linnaean or phylogenetic species), approximately 38% of South African species on the checklist have been barcoded. Only 5,232 (58%) unique species names could be assigned to these BINs. The remaining 42% point to gaps in taxonomic knowledge highlighting the need for further taxonomic research, Notably, 1,730 (~19%) species names represent taxonomic discrepancies because their barcodes resulted in BIN merges (due to low interspecific variation) or splits (high intraspecific variation).

**Table 1:** Summary statistics of South African taxon representation mined from BOLD on 19 October 2023. These data are for animals (*COI*), fungi (ITS), and plants (*matK* and *rbcLa*). BINs - Barcode Index Numbers; BOLD – Barcode of Life Database Systems; SA – South Africa.

Taxonomic group	Estimated No. of known species on SANBI checklist	SA species in BOLD	% of SA species in BOLD	No. of BINs	BIN to species ratio	No. of BIN merge/ total BIN merge records	No. of BIN split/ total BIN split records	Specimen without BIN no.	No. of unpublished BOLD records	No. of published records in BOLD
Amphibians	134	49	36.6	84	1.7	0/ 0	5/ 12	98	301	430
Annelids	1,024	48	4.7	166	3.5	1/ 2	6/ 14	203	214	925
Arachnids	5,046	491	9.7	2,279	4.6	34/ 78	99/ 279	8,741	13,466	20,139
Birds	862	176	20.4	193	1.1	10/ 20	9/ 19	31	656	616
Cnidarians	1,214	13	1.1	15	1.2	0/ 0	1/ 3	14	288	52
Crustaceans	2,088	84	4.0	147	1.8	1/ 2	9/ 26	393	1,542	1,144
Echinoderms	483	98	20.3	120	1.2	5/ 14	12/ 38	226	191	831
Fish	5,890	1,012	17.2	1,355	1.3	26/ 131	50/ 111	446	4,722	8,280
Fungi	12,581	349	2.8	0	N/A	0/ 0	0/ 0	1,023	373	947
Insects	36,649	2,754	7.5	28,280	10.3	90/ 216	247/ 590	82,689	371,835	417,069
Mammals	345	99	28.7	101	1.0	10/ 20	16/ 36	282	1,696	836
Molluscs	1,696	183	10.8	363	2.0	3/ 6	22/ 56	277	2,074	2,013
Plants	21,524	3,469	16.1	119	0.0	0/ 0	0/ 0	20,461	9,927	12,990
Platyhelminthes	88	24	27.3	17	0.7	0/ 0	0/ 0	157	11	215
Reptiles	406	55	13.5	44	0.8	1/ 2	5/ 11	140	478	267
Sponges	378	1	0.3	9	9.0	0/ 0	0/ 0	2	25	48
*Other Animals	1,879	60	3.2	1,245	20.8	0/ 0	4/ 8	15,514	32,923	22,297
*Other Organisms	N/A	99	N/A	123	1.2	5/ 11	8/ 25	552	897	758
**Total**	**92,287**	**9,064**	**9.8**	**34,660**	**3.8**	**186/ 502**	**493/ 1,228**	**131,249**	**441,619**	**489,857**

* A list of taxa under the taxon groups “Other animals” (Acanthocephala, Ascidiacea, Bryozoa, Chaetognatha, Chilopoda, Collembola, Ctenophora, Diplopoda, Diplura, Gastrotricha, Myxini, Nematoda, Tardigrada) and “Other Organisms” (Apicomplexa, Bacteria, Chromista, Pinophyta, Pyrrophycophyta).

BINs are based solely on *COI* sequences and are therefore not applicable to fungal ITS and plants *matK* and *rbcLa*.

**Fig 1:**
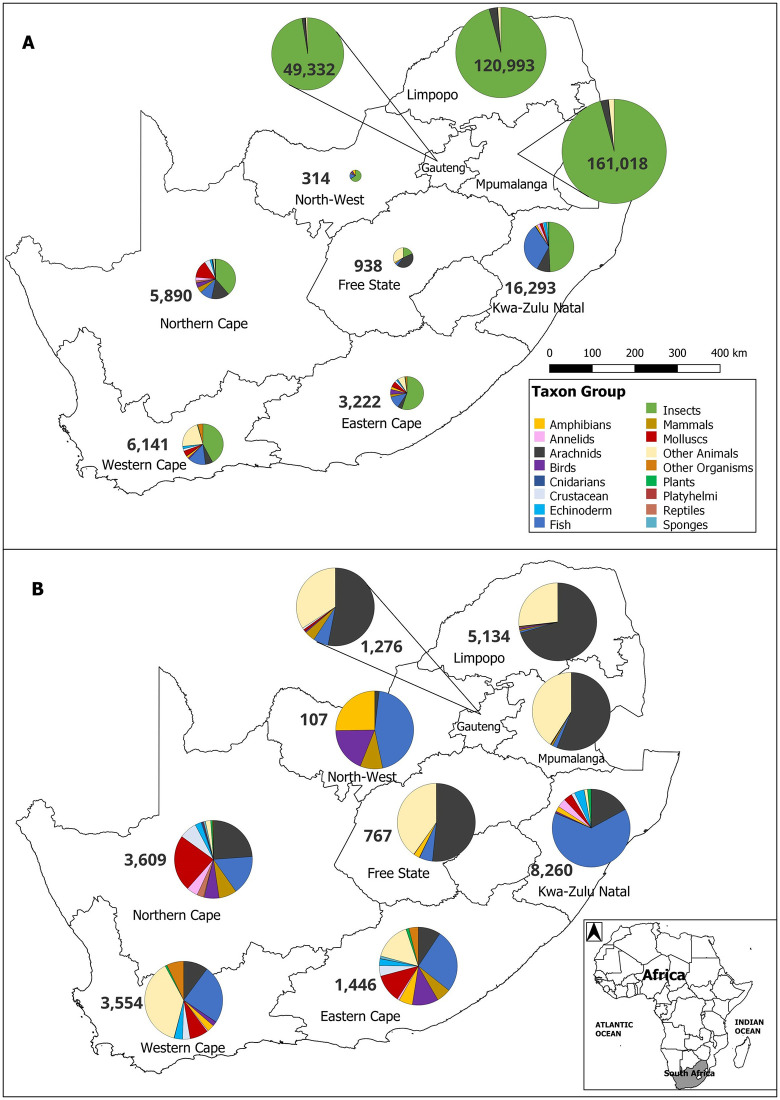
Representation of taxon groups with BINs in each South African province. Taxon group ‘Fungi’ is excluded because it had no BIN records on BOLD. Category “Other Animals” are species that do not fit into the outlined taxon groups. Category “Other Organisms” are organisms other than animal, plant or fungi (e.g., Protista). Numbers indicate the number of BINs. **A.** Proportional representation including all taxon groups. **B.** Proportional representation excluding ‘Insects’. Map generated in QGIS using species occurrence data from BOLD (https://boldsystems.org) and shapefiles from DIVA-GIS (https://diva-gis.org/data.html) and GADM (https://gadm.org). Figure created anew for this study for comparison with da Silva and Willows-Munro (2016) [34].

Furthermore, the 3,832 unique names not assigned to BINs likely reflect non-compliance with iBOL barcode standards or missing sequence data for the corresponding specimens. Insects had the highest number of records (417,069; 84%; Fig 1A) and the highest proportion of BINs (334,731; 91%), but only 9% were assigned to nominal species. Amphibians were the best-represented taxon, with 36.6% of all known South African species *COI*-barcoded, followed by *COI* barcodes for mammals (28.7%), platyhelminthes (27%), Birds (20.4%), Echinoderms (20.3%), fish (17.2%) and reptiles (13.5%). Representation of the remaining animal groups varied (Table 1). Overall, insects, annelids, arachnids, cnidarians, crustaceans and molluscs had relatively lower representation (1–11%). Sponges were the least barcoded taxon, with only one associated species name on BOLD out of 378 on the South African species checklist (48 records were associated with 9 BINs). Fungi are underrepresented (2.8%), while plants with the unique two-gene marker system (*matK* and *rbcLa*) on BOLD could not be fully captured in our analyses. It is important to note that the Barcode Index Number (BIN) system was developed specifically for clustering animal *COI* sequences and is not compatible with plant barcodes (matK and rbcLa) or fungal ITS regions [15]. Furthermore, despite plants having relatively high species count, the low 119 BINs highlight the incompatibility of the BIN system to standard plant barcoding markers.

### The most common genetic markers used for generating South African data available on GenBank

*COI* was the most used marker for more than half of the 16 taxa analysed (S5 Table). The second most frequently used marker varied across groups: *cytb* in annelids and fish; *16S* in crustaceans, echinoderms and molluscs; *28S* in cnidarians and insects; and *LSU* in sponges. The third most frequently used markers were *5.8S*, *mutS*, *18S*, *EJP9*, *D-loop*, *EF1A*, *ITS2*, and *28S* in the respective groups. Six taxonomic groups had more published records for gene markers other than *COI* including genome sequence data: amphibians (*16S*), arachnids (Whole Genome Shortgun Sequence – *WGSS*), birds (transcript variant X), mammals (*WGSS*), flatworms (*28S*), and reptiles (*cytb*). For these taxa, the second most common markers were *12S*, transcribed RNA, *cytb*, cDNA clone, *ITS2*, and *ND4*. The third most used markers in these groups were *ND2*, *COI*, *ND3*, transcript variant X, *5.8S*, and *16S*. Notably, *COI* was only the fourth most used marker in flatworms and fifth in birds, and was less used in amphibians, reptiles, and mammals (all vertebrate groups). For plants, the most used marker was *5.8S*, followed by *rbcLa* and *matK*. For fungi, *WGSS* and other ITS markers (*5.8S*, *ITS2* and *ITS1, LSU* and *SSU*) were the most frequently-used. It was noteworthy that for arachnids, birds, fungi, and mammals, High-Throughput Sequencing (HTS: advanced DNA sequencing methods that allow for sequencing millions to billions of DNA fragments simultaneously) records appear to represent the most utilised sequences, moreover, HTS sequences, particularly WGSS, were treated as a single marker to enable comparisons with other markers. However, these records do not meet the criteria for DNA barcoding nor can they be used as a standard identification sequence and are therefore only highlighted and will not form part of the analysis of the three most-frequently used gene markers.

### Geographic coverage of South African BOLD data

The total number of barcode-compliant records (assigned to BINs) with geographic information was 367,354. The largest contributors were Mpumalanga (44%), Limpopo (33%), and Gauteng provinces (13%). The coastal provinces contributed far less to the reference library: KwaZulu-Natal (4.4%), Western Cape (1.7%), Northern Cape (1.6%), and Eastern Cape (0.9%). The contributions from North-West and Free State were negligible (less than 0.01%). Among taxonomic groups, insect barcodes dominated the contribution of BINs to BOLD across all nine provinces (Fig 1) and represented more than 30% of the BINs generated for each province. The exception was the Free State, where arachnids were predominant (42.2%). In Mpumalanga, Limpopo and Gauteng, insects represented more than 95% of all barcoded species and contributed the highest number of BINs (90%). Insects also formed the largest proportion of barcodes for the four coastal provinces: Eastern Cape (55.1%), KwaZulu-Natal (49.3%), Western Cape (42.6%), and Northern Cape (38.7%). With respect to vertebrates, fish were the most barcoded among provinces, with most of the records coming from KwaZulu-Natal (71%), followed by Western Cape (12%), Northern Cape (8%), Eastern Cape (5%), Mpumalanga (2%), and Gauteng (1%). North-West, Limpopo, and Free State contributed fewer than 1% of fish records. The majority of plant records with an assigned BIN number came from KwaZulu-Natal (63%), followed by Western Cape (14%), Northern Cape (14%) and Eastern Cape (9%). Within the marine environment, fishes accounted for most of the records (3,781), followed by echinoderms (597), annelids (509), sponges (46), cnidarians (30), and platyhelminths (14).

### Institutions contributing to South Africa’s DNA barcode library

The barcode records from South African localities available on BOLD were contributed by 194 institutions across 32 countries, including South Africa (Table 2). Universities were the leading contributors, predominantly based in Europe. The Centre for Biodiversity Genomics (CBG) at the University of Guelph (Canada) contributed 86.2% of DNA barcode records (S1 Fig), due to its role as the sequencing facility for collaborative projects (e.g., BIOSCAN [42]) with South African researchers. In contrast, South African institutions directly contributed to only 8.9% of the barcodes. However, as BOLD and GenBank share data, our analysis indicates that the number of sequences submitted and published for specific taxa or gene regions is substantially higher on GenBank than the number of sequences imported from GenBank and published by BOLD. This is because barcode sequence data is submitted directly to GenBank without being deposited in BOLD first or in parallel. This results in many records on BOLD not having GenBank accession numbers (S3 Fig). For South African records, GenBank contained 42 times more fungal sequences, 5 times more plant sequences, and 3.5 times more animal sequences than BOLD had mined. This disparity was probably due to GenBank’s primary role as a repository for the deposition of sequences, including DNA barcodes, for long-term archival purposes, which is often a requirement for journals when publishing sequence data. However, many records from GenBank lack metadata, such as locality, collection date and images [43] affecting the usability of the data, particularly with regards to the verification of identification, genetic changes over time and genetic variation distribution [44–46]. While GenBank and BOLD databases share data, BOLD requires that data submitted directly to their database, should meet minimum Darwin core standard requirements.

**Table 2:** Breakdown of the world regions and institutions that contributed to South African specimen records on BOLD Systems. Ni – Number of institutional or individual contribution; Nr – Number of specimen records submitted to BOLD.

Country/ Region	Museums		Research Centre		Universities		Private Research Collections		Government Department		Uncertain		Total no. of specimens on BOLD
	Ni	Nr	Ni	Nr	Ni	Nr	Ni	Nr	Ni	Nr	Ni	Nr	
South Africa	9	1,714	8	12,876	11	21,342	7	479	0	0	0	0	**36,411**
Africa (Kenya, Nigeria)	0	0	1	8	1	29	2	79	0	0	0	0	**116**
South America (5 countries)	1	27	3	15	1	1	0	0	0	0	0	0	**43**
North America (USA, Canada)	7	142	5	14,644	18	431,056	5	28	8	307	0	0	**446,177**
Australia	3	25	4	33	3	30	0	0	1	2	0	0	**90**
Europe (17 countries)	20	1,415	22	4,087	31	931	6	252	0	0	8	78	**6,763**
Asia (3 counties)	0	0	2	4	1	2	2	8	0	0	0	0	**14**
Middle East (Israel)	0	0	1	9	1	1	0	0	0	0	0	0	**10**
No Institution	0	0	0	0	0	0	0	0	0	0	0	233	**233**
Total	40	3,323	46	31,676	67	453,392	22	846	9	309	8	311	489,857

A full list of countries not captured on the table in alphabetical order: Argentina, Austria, Belgium, China, Colombia, Croatia, Czech, Denmark, Finland, France, Germany, Italy, Kyrgyzstan, Lithuania, Mexico, Netherlands, New Zealand, Norway, Peru, Russia, Spain, Switzerland, United Kingdom, Unnamed Countries.

### Taxonomy-related publications using DNA barcodes

**Fig 2:**
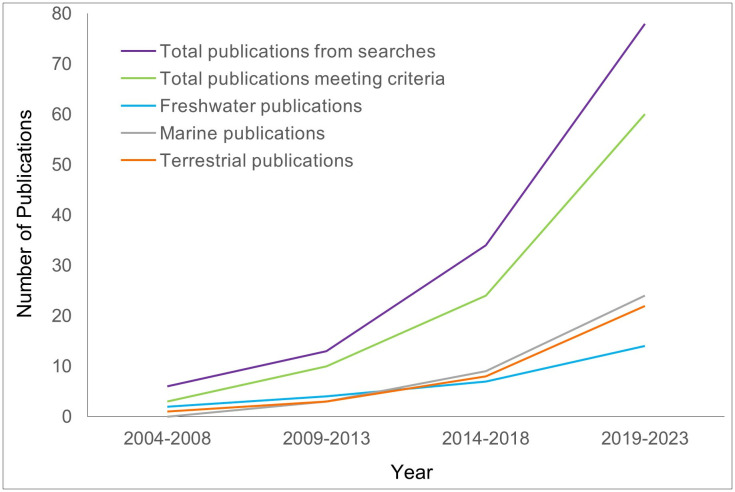
Publications on taxonomic revisions, descriptions, and checklists of South African taxa related to DNA barcoding per habitat over time.

**Fig 3:**
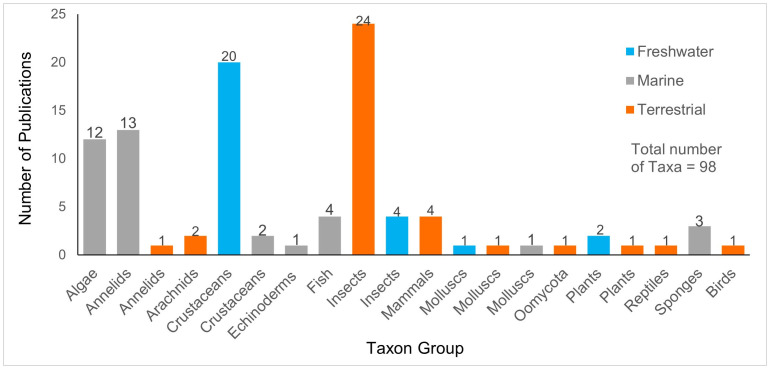
Number of taxa with DNA barcodes used in taxonomic publications, categorised per taxon group and habitat in South Africa over the 19-year period (2003–2023) that was analysed.

The number of peer-reviewed taxonomic revisions, descriptions, and checklists of South African taxa related to DNA barcoding and published between 2003 and 2023 was 98, averaging five publications per year, with over 87% of publications recorded between 2014 and 2023 (Fig 2). Categorising publications into terrestrial, marine or freshwater realms showed that the use of DNA barcoding in taxonomy occurred earlier for terrestrial and freshwater species than for marine species, with the number of marine publications increasing from 2014 and now surpassing those of both terrestrial and freshwater realms (Fig 2). Over the 19-year period that was analysed, terrestrial insects and freshwater crustaceans accounted for most publications (24.4% and 20.6%, respectively), followed by marine annelids (13.4%), and marine macroalgae (12.4%). The remaining taxon groups were each represented by one to four publications (Fig 3).

## Discussion

### Progress towards filling the taxonomic and geographic DNA barcoding gaps over the past 10 years

The use of *COI* as a standard for animal species identification has increased significantly in South Africa since Hebert et al. (2003) [12] introduced the concept of ‘*DNA barcoding*’ in 2003. The following year, 543 records, mainly fish (99%), were deposited on BOLD through the South African Institute of Aquatic Biodiversity (SAIAB). By 2008, prior to the introduction of the plant DNA barcoding standard (*matK* and *rbcLa*) proposed by Hollingsworth et al. (2009) [19], South Africa had already contributed 346 more records to BOLD through the University of Johannesburg. Furthermore, South Africa had already begun sequencing and uploading ITS markers to BOLD in 2008, through Agriculture and Agri-Food (agricultural department of Canada) and the Centraalbureau voor Schimmelcultures (Royal Netherlands Academy of Arts and Sciences [KNAW] – Westerdijk Fungal Biodiversity Institute), prior to Schoch et al. (2012) [20] formally proposing ITS as the standard DNA barcode for fungi. Scientific output in terms of publications, collaborations and records on BOLD, shows that DNA barcoding research in South Africa is thriving.

As of 19 October 2023, South African records totalled ~0.5 million, with animals accounting for 97% of the records, whereas plants made up 2.6%, fungi 0.2%, and other organisms 0.2%. Since the da Silva and Willows-Munro report in 2016 [33], animal records have increased from 48,000–475162 (890%), of which insects have increased the most from 37,105–417,069 records (1024%). BOLD records without assigned BINs represent 26.8% of all records, suggesting that the associated sequences were either not barcode-compliant or that these records were not yet sequenced with the approved iBOL markers, although the metadata for the specimen were loaded.

A total of 127,363 (26%) South African BOLD records could only be assigned at higher level classification (order, class, phylum, kingdom), lacking identification at the species, genus and family levels (Table 1). The remaining 74% (362,494) of records had identification to family, of those 102,625 were identified to genus and of those identified to genus 53,213 were identified to species level. Consequently, most South African records remained inadequately identified. A decline in taxonomic identification could explain this, or that the quantity and diversity of barcoded specimens surpassed the available expertise and capacity. Among the unique records identified to species level (9,064), most are animals (7432) with the majority being insects, representing an increase from the previous number of records reported by Myburgh et al. (2021) [47]. In contrast, by 2023, far fewer records were identified to species level in plants (1360), fungi (181), and other organisms (91).

Insects represent the highest percentage of all BOLD records identified to species level, due to the high number of records in comparison to other taxonomic groups. Upon closer examination, insects have the lowest percentage (7.7%) of records with a species name of all 417069 records. This might be due to the ease with which many species could be collected in *en masse* using methods such as Malaise traps [48]. However, this leads to the collection of many hyper-diverse micro-insect species, recently termed “dark taxa”, for which using traditional taxonomy to identify to species level is challenging [49]. Additionally, the presence of invasive or cryptic species among the collected specimens may affect the analysis, where the order Psocodea is represented by a single species on the checklist but shows 16 species and 180 BINs in BOLD. Furthermore, Psocodea has unresolved taxonomic issues of the now-deprecated orders Pscoptera (barklice) and Phthiraptera (lice) [50,51]. Therefore, using BOLD to identify these species is difficult due to the incomplete reference library and the checklist that is incomplete [52]. It therefore becomes essential to close the reference library gap, where a better understanding of the dark taxa would be possible through the BIN system which clusters similar DNA sequences into OTU, allowing minimal reference to be made such as OTU spatial distribution [43,53,54]. However, until such an OTU (BIN) is given a formal taxonomic name, it will be known by its OTU and have limited functionality.

Although our study reveals a significant increase in South African records on BOLD since 2016, there is evident need to address gaps in neglected taxonomic groups, improve record quality, and enhance taxonomic expertise. The categorisation of BOLD records into 18 taxon groups revealed that eight of these represent less than 10% of the estimated number of known species in South Africa, while nine groups had representations ranging from 10% to 37%. This emphasises the importance of expanding DNA barcoding within all taxon groups. Lopez-Vaamonde et al. (2021) [55] demonstrated the significance and utility of having a comprehensive barcode library for a taxon group. By barcoding and verifying 91% of Gracillariidae (Lepidoptera) in Europe, the reference library was greatly improved. To achieve similar progress, efforts like those undertaken by Raphalo et al. (2021) [56], Kamdem et al. (2023) [57], and Botha et al. (2023) [58] of barcoding new species need support, including barcoding species for which there are no BOLD records. Intentional barcoding of new species is crucial not only for increasing the representation of each taxon group relative to the estimated number of known species in South Africa but also for bringing the reference library for each taxon group closer to completion.

A comprehensive reference library (i.e., a repository or database containing sequences derived from specimens identified by researchers or taxonomic experts [59]) requires high-quality records. The reference library should facilitate species recognition and recovery of species boundaries, where feasible [60]. Ideally, at least one record for each BIN should be linked to a specimen identified to species level. The record should include specimen images and occurrence data (e.g., the GPS coordinates of the locality where the specimen was collected), and the taxon should be sequenced using a standard marker for that group. Additionally, the sequences should consist of two consensus sequences of appropriate length, depending on the species, or have less than 1% ambiguous bases [60].

Oliveira et al. (2016) [61] and Fontes et al. (2021) [62] proposed that a species should have a minimum of three sequences to make a meaningful contribution to the reference library. Of the almost 0.5 million South African records on BOLD, 9% contain BINs and are identified to species level, representing 5,232 unique species. However, most taxon groups contain species with one to four sequences that meet the barcode standard. We did not analyse other types of data that enhance sequence quality, such as images, consensus sequences, base count, or base ambiguities. We looked only at the number of sequences that meet the barcode standard per species. However, we conclude that generating higher-quality sequence records will be more valuable than producing large numbers of lower-quality sequences.

Definitive identification of specimens to species level plays a crucial role in the creation of a comprehensive DNA barcoding reference library [63,64]. This is especially true for newly identified species or specimens that do not return a close match to existing records [65]. Table 1 and S4 Fig highlight a high BIN-to-species ratio, which suggests the presence of high *COI* diversity or, alternatively, unnamed/cryptic taxa. Hence, taxonomists must identify such specimens following conventional taxonomic processes [48]. The percentage each level of taxonomic classification category contributing towards the data of each taxonomic group were above 50% at a species level for most taxon groups. However, Arachnids, insects, other animals, other organisms, plants and sponges have less than 50% of their records bearing species identifications. These observations underscore the need for continued taxonomic verification and curation to ensure the reliability of DNA barcode reference libraries, particularly for taxonomically diverse or poorly documented groups.

The need for more taxonomists cannot be overemphasised, as their expertise is essential to address this growing knowledge gap. South Africa is fortunate to have several experienced and internationally recognised taxonomists in the terrestrial, freshwater and marine realms who have been contributing to the enrichment of the reference library [66–70].

The South African government has allocated funding towards projects that encourage the development of natural scientific expertise, including taxonomy, such as the Natural Science Collection Facility (NSCF), the FBIP, National Research Fund (NRF) Professional Development Program Postdoctoral Fellowships and Royal Museum for Central Africa (RMCA, Belgium - https://www.africamuseum.be/en). Through these initiatives, new taxonomists are expected to be trained and contribute to a variety of specialised subjects, including marine and freshwater biology. This emerging group of specialists ensures that South Africa remains at the forefront of taxonomic research, supporting a better understanding and preservation of the country’s diverse biological heritage.

The quality of spatial data is one of the key factors contributing to a good DNA barcode record [12,21]. Most South African records with BINs contain metadata allowing the geographic mapping of the specimen. In eight out of nine provinces, most records are from insects. This is possibly the result of insect collection methods that are often easy, non-targeted, and result in mass captures [47,48]. However, it is surprising that some taxon groups that may be considered charismatic, do not appear in some provinces: *COI* records of reptiles seem to be missing in six provinces (Eastern Cape, Free State, Gauteng, KwaZulu-Natal, North-West, and Western Cape), mammals in the Free State, and amphibians in Gauteng. These are well-known and well-studied taxon groups for which DNA barcoding of at least one species could have been achieved for all provinces. A number of other taxon groups are missing in each province (S6 Table), highlighting the spatial gaps that need to be filled. It is important to highlight that only public records with assigned BINs were considered. Fungi were excluded because no BINs were assigned to them. In contrast, plants were included because BINs have been assigned, largely derived from historical experimental sequencing projects, mitochondrial genome studies, or GenBank-imported data using the *COI* gene, which is not the standard barcode marker for plants [71]. Additionally, it is noteworthy that for certain taxonomic groups, particularly vertebrates, different genes are preferred over *COI* [72], leading to low spatial coverage due to the limited number of records. Accessing all South African data from BOLD, including private data, may help to obtain a clearer picture of the spatial distribution of barcoded specimens.

As climate impacts biodiversity [73,74], it has become imperative and urgent to document biota. Africa, with many developing countries [10], already struggles with this task and will have to find effective ways to meet this Convention on Biological Diversity (CBD) obligation. This may include allocation of governmental funds, partnering with local and international stakeholders, taking advantage of technological advancements, sourcing funds from international stakeholders, and undertaking capacity development and skill transfer in the biodiversity sector. The South African government provides several annual funding opportunities, and the private sectors also contribute significantly to biodiversity research, e.g., the Jennifer Ward Oppenheimer Research Grant. The Global Environment Facility (GEF) offers funding to developing countries to support them in fulfilling their environmental commitments. The Jacob Richard Schramm (JRS) Biodiversity Foundation and the Rufford Foundation provide small grant opportunities for conservation research. Additionally, funding opportunities may sporadically arise from governments, Non-Governmental Organizations (NGOs), and the private sector. Maintaining connections with various stakeholders in the biodiversity sector is essential for staying informed about emerging funding streams and potential collaborations.

BOLD, GenBank and the European Molecular Biology Laboratory (EMBL) are among the most widely used bioinformatics databases for attempting species-level identification [75,76]. GenBank, the largest and most used of the databases, and EMBL serve as repositories for genetic sequences [34,36]. BOLD has comparatively stricter requirements and serves as a repository for sequences that meet DNA barcoding standards. In this study, sequences uploaded on GenBank that meet the BOLD requirements were mined by the latter database, through a data-sharing agreement established between BOLD and GenBank [13]. The total number of South African sequence records on GenBank that used the barcoding markers required by BOLD and had subsequently been mined, was 13,722 (animals – 9,804 [*COI*], fungi – 859 [ITS], plants – 3,059 [*matK*, *rbcLa*]). In contrast GenBank contained over 86,345 South African sequence records for the same markers in animals (34,613), fungi (36,625), and plants (15,107). The total sequence records mined by BOLD from GenBank were relatively low (16%). The proportion of mined animal sequences (28% of the total) showed a significant increase to 85% from the 15% previously reported [33].

GenBank contains numerous records for additional DNA sequencing markers that are invaluable for species identification. The top three markers for each taxonomic group are highlighted in the present work (S5 Table), which can be considered optimal for use alongside standard DNA barcoding markers to enhance accurate species identification. A multi-locus approach is widely regarded as the most effective method for species identification [77,78]. The GenBank data analysis also highlights taxonomic groups where DNA barcoding markers are not among the three most frequently used regions (S5 Table). For example, *COI* ranks ninth and tenth for amphibians and reptiles, respectively. Incorporating DNA barcodes with other sequences will help to update and maintain the reference library. In other groups, such as arachnids and mammals, *COI*-containing mitogenomes, whole genomes, and other high-throughput sequencing (HTS) products have become standard benchmarks. However, these typically do not meet the technical criteria established for DNA barcoding.

Our analysis of the most widely-used gene markers may also suggest the limitations of *COI* in species identification. These limitations could involve technical issues such as failure in PCR amplification and sequencing or could be biological in nature such as species hybridisation, or the difficulty in resolving species boundaries within very recently-evolved or rapidly-radiating taxon groups. Several studies demonstrated that *COI* produced fewer sequences or lower species identification accuracy compared to other gene regions such as *16S* rRNA for some taxon groups [79,80], whereas, Lv et al. (2014) [81] found no significant difference in the performance of *COI*, *16S* rRNA, *ITS2*, and *12S* rRNA in identifying Ixodida tick species. These studies emphasised that *COI* data alone are insufficient for achieving accurate species identifications, especially when using a single-gene approach. As a result, researchers may opt for alternative genomic regions beyond the standard barcoding markers for more accurate identification.

In South Africa, steps have been taken to fast-track the gathering of biodiversity information to better implement conservation strategies [31,82]. National and international stakeholders have been mobilised in a collaborative effort to fill the gaps in the reference library on BOLD. At the national level, South Africa has established SABOL to facilitate and promote DNA barcoding research, with the goal of addressing gaps in the reference library, among other objectives. South Africa had directly contributed 16,861 DNA barcoding records to BOLD from 26 institutions as of 2015 [33]. By 2023, this contribution increased by an additional 19,550 records from 35 institutions, accounting for approximately 54% of the total records to date, with a greater number of institutions participating over a shorter time frame, making it the second-largest contributor (7.4%; Table 2). The highest contribution is from the CBG, with over 428,797 (86.2%) records (S1 Fig). Collaborative projects, including those under BIOSCAN, such as Malaise trap sampling in Kruger National Park, facilitated the collection of specimens that were sent to the Centre for Biodiversity Genomics (CBG) for DNA barcoding and subsequent submission to BOLD. As a result, Canada emerged as the largest contributor of South African DNA barcoding records on BOLD. Other contributors originate from across the globe, with combined contributions from Asia (three countries), Australia, Europe (17 countries), the Middle East (Israel), South America (five countries), North America (USA), and Africa (Kenya and Nigeria) amounting to 25,640 (5%) records. This indicates the importance of collaborations nationally and internationally to meet national objectives on biodiversity conservation.

Long-term government funding for biodiversity research has a high impact in developing the necessary capacity, skills, and infrastructure to fast-track biodiversity description and, ultimately, conservation. The South African governments established multiple long-term funding streams through the Department of Science and Innovation (DSI) for biodiversity research, capacity development and skills transfer as part of the South African Research Infrastructure Roadmap (SARIR) and directly as the FBIP. Through the FBIP, the government has directly awarded more than US$3.4 million to over 169 biodiversity research projects between 2013 and 2022. Of these, 124 projects amounting to US$3.2 million included genetic analyses, permitting the production and contribution of DNA sequences to databases like BOLD and GenBank. The FBIP and SARIR have broad components advocating for capacity development, skill transfer, and succession planning within and for future biodiversity research [32,83]. Large grants awarded by the FBIP require that post-doctoral and young researchers take part, as can be seen in the Eastern Cape Forest Project, BioGaps, REFRESH, SeaKeys, and SeaMap projects. The SARIR projects, such as the Natural Science Collections Facility (NSCF), address capacity and skill shortages in collection facilities and set out plans for human development [82]. The SABOL offers training and capacity development in DNA barcoding and advanced related approaches like eDNA (environmental DNA: DNA shed by organisms into the environment), sampling, and metabarcoding.

### Advances and applications of DNA barcoding: Case studies of implementation in various fields

DNA barcoding has advanced significantly since its introduction, particularly with the development of HTS, which enables rapid sequencing of numerous DNA fragments simultaneously [84]. New techniques that utilise HTS, such as metabarcoding of eDNA, have emerged as powerful tools for large-scale biodiversity monitoring, as multiple species can be identified simultaneously [85,86]. Since the first assessment of the status of DNA barcoding in South Africa in 2016 [33], research has started to incorporate HTS. Multiple institutions now possess HTS equipment focused on biodiversity monitoring and research. When combined with a comprehensive reference library, these methods enhance data quality, and their applications in biodiversity research are broadened.

The application of HTS in the marine environment in South Africa has been gaining momentum [86,87], in particular, the metabarcoding of zooplankton. Singh et al. (2021) [87] used metabarcoding to determine the species composition of plankton from samples collected with tow nets along the east coast of KwaZulu-Natal (Western Indian Ocean) and identified 45% of the samples to species level on BOLD. Oosthuizen et al. (2023) [5] and Govender et al. (2023) [88] later used metabarcoding in zooplankton in the Northern Cape (Atlantic Ocean) and KwaZulu-Natal (Indian Ocean), respectively. Oosthuizen et al. (2023) [5] highlighted gaps in the current marine zooplankton reference library for the Northern Cape province, as many OTUs could be assigned only to higher taxonomic levels. They revealed minimal overlap between the results of eDNA analysis and the species checklists of known taxa in the Atlantic region of South Africa. On the other hand, Govender et al. (2023) [88] was able to match 63% of the sequences from KwaZulu-Natal to species, with many complementing the more comprehensive DNA barcoding checklists from the region.

Furthermore, the application of HTS approaches is increasingly evident in studies of South African freshwater systems. Research focused on eDNA assessments of freshwater systems has demonstrated the advantages of metabarcoding. For example, a study assessing eDNA of freshwater fish in estuaries within the eThekwini Municipality (KwaZulu-Natal province) was able to successfully identify and match 54% of the OTUs to a reference sequence [89]. Mashaphu et al. (2023) [90] demonstrated that within a checklist of known native freshwater fish species 34 species, six genera but no families lacked *COI* reference sequences, while 86 species, 22 genera, and eight families lacked *12S* reference sequences. These studies emphasise the critical role of a comprehensive reference library, especially in the context of developments such as HTS.

DNA barcoding also has applications in wildlife forensics [24,26,91]. The South African government, through the Department of Forestry, Fisheries and the Environment (DFFE), has collaborated with the National Prosecuting Authority (NPA), the South African Police Service (SAPS), South African Institute for Aquatic Biodiversity (SAIAB), African Centre for DNA Barcoding (ACDB), SANBI, and iBOL to implement a national DNA barcoding reference database using the BOLD Systems [92]. This database will be populated with DNA barcodes from samples collected under chain of custody, following SAPS-approved protocols, and used as a reference database in wildlife crime investigations for species identification, among other uses. The identification of suspect material will inform subsequent law enforcement actions, further highlighting the novel applications of DNA barcoding reference libraries in South Africa. Additionally, DNA barcoding has already been applied in medicolegal forensic entomology in South Africa and globally [93,94], and has also found application in the quality assurance of fly larvae being used in medical maggot debridement therapy [95].

### Key challenges and opportunities of DNA barcoding in South Africa

The use of DNA barcoding presents many opportunities to assist biodiversity conservation. Its ability to rapidly and reliably identify specimens and make the data accessible to relevant authorities enables fast response times. The value of this information is enhanced when supported by well-curated specimens that can be easily accessed for verification. However, many metabarcoding studies proceed despite the limited availability of reference sequences needed for the validation of accurate species identification. As a result, the traditional taxonomic approach to identifying unknown or unmatched specimens is disabled.

For a long time, various institutions involved in the biodiversity conservation value chain operated in isolation. However, government initiatives, through the SARIR, such as the NSCF, DIPLOMICS, the FBIP, and the Biodiversity Biobanks South Africa (BBSA) have facilitated collaboration, enabling institutions to find common ground and establish networks [31,82]. The BBSA (https://bbsa.org.za/) aims to expand the diversity and quality of stored samples while enhancing accessibility for research and development. DIPLOMICS (https://www.diplomics.org.za/about-us) aims to strengthen omics capacity, advance research quality, and expand access to omics technology and expertise, supporting the “1KSA” biodiversity genome sequencing program to generate draft genome assemblies from voucher-supported specimens, thereby enhancing DNA barcoding and providing a foundation for future population genomics research. The objective of these programmes is to establish an integrated science and infrastructure facility that links DNA barcodes to their corresponding tissue samples, which are stored in biobanks, and to specimens preserved in museum collections. Both the biobank and the museum are responsible for the long-term storage and curation of these samples, which can be accessed at any time for sequence (barcode) verification or species name validation when necessary.

Strengthening collaboration between data generators and end users enhances the value and application of DNA barcoding [31]. Despite its potential, many biodiversity-related sectors remain unaware of how to effectively use barcoding data. DNA barcoding offers significant advantages for rapid and accurate species identification, particularly in spatial planning and land-use management, where timely identification of specimens collected during Environmental Impact Assessments can support the conservation of threatened species and inform development decisions. In addition, agriculture would benefit from identifying pests in their early stages of infestation [96]. Furthermore, accurately differentiating CITES-listed species from their look-alikes will help to manage illegally traded species, and early detection of alien species may help to prevent future invasions. Therefore, promoting cross-sectoral collaborations, where researchers using molecular tools are involved in interpreting barcoding data for policy and decision making, will enhance the adoption of DNA barcoding. Additionally, support from both local and international scientific communities strengthens capacity and technological transfer that can increase the credibility of library creation. This is exemplified by the collaboration between various regions, government departments and iBOL in generating specific datasets, as discussed in this paper.

DNA barcoding holds significant potential, although the costs (field sampling, laboratory resources, sequencing) can be a limitation, especially in developing countries. While most barcoding reviews have not addressed the costs of building a library, they have highlighted HTS as a means to reduce expenses [33,84,86]. Studies have estimated the cost of Sanger sequencing to be US$5–$15 per sample [97,98], whereas optimised HTS workflows could lower these costs to US$0.10 [99,100]. HTS is generally more cost-effective, as it can detect multiple species in one sample, further reducing the cost per species identified compared to the single-species (sample) approach of Sanger sequencing. As of 2024, Sanger sequencing (bidirectional) costs among South African facilities ranged from US$28.00 to $29.40 per sample [101]. Despite sequencing costs appearing high in South Africa, HTS ultimately offers significant savings per species, but this is mostly for large-scale barcoding efforts and is not necessarily cost-effective for small-scale projects [27]. a major limitation of HTS is the large volume of data it generates [102], which raises two challenges related to infrastructure and technical expertise [83]. Effective analysis requires significant storage space, specialised software, and high computational power. Cloud-based platforms are often used as a solution to address these demands [86,100]. Selecting the appropriate analytical software is also challenging, as commercial packages are costly, whereas open-source alternatives require programming skills [102]. These contains are further compounded by limited bioinformatics, highlighting the need for targeted investment in training and capacity development for biodiversity research [42,86,99].

Another major challenge of the eDNA and metabarcoding approach is the inability to detect BIN discordance. This is because sequences cannot be matched to individual voucher specimens to verify the accuracy of BIN assignments [42,48]. The potential for overestimation, and possibly underestimation, of species numbers using these methods is real but, in the absence of a fully comprehensive reference library with multiple records per species, cannot be measured or even estimated using current procedures [103]. For reliable estimation of species diversity via eDNA and metabarcoding to become a reality, species coverage of the barcode reference library needs to be vastly increased. Furthermore, challenges associated with analysing plant data on BOLD should be addressed, particularly as the database employs a two-locus system for plant DNA barcoding (*matK* and *rbcLa*) as recommended by the Plant Working Group [19,27]. However, for BOLD to assign BINs to a sequence, it must meet set requirements, including representation by a single locus, a requirement that the experimental *COI* gene in plants did not meet. It is therefore crucial that the criteria for assigning BOLD BINs to plants be established to fully leverage the potential benefits of the BIN system.

The standard DNA barcoding regions recognized by iBOL (*COI*, *matK*, *rbcLa*, ITS) are not universally applicable across all taxonomic groups, resulting in challenges related to amplification success and sequencing quality [104]. The *COI* gene region is widely used for animal DNA barcoding because it is relatively conserved within species but variable between species [17]. However, its effectiveness is not universal across all taxa; for example, the Folmer region often performs poorly in amphibians [105]. In such cases, alternative markers, including *Cytb*, *12S*, and *16S*, are used to complement or replace *COI* [80]. In plants, *matK* and *rbcLa* are commonly employed, though *rbcLa* amplifies easily but poorly discriminates species, while *matK* discriminates well but is difficult to amplify, especially in non-angiosperms; alternative chloroplast spacers such as *trnH-psbA*, *atpF-atpH*, and *psbK-psbI* are sometimes used [106]. For fungi, ITS is the primary barcode, with *TEF-α*, *RPB*, and *β-tubulin* serving as alternative markers when necessary [107].Efforts should be directed toward developing more effective primers, ideally within the standard DNA barcoding gene regions, to enhance the applicability of DNA barcoding across diverse taxonomic groups.

### Current knowledge gaps to prioritise for future efforts

We conducted a gap analysis of the DNA barcoding reference library of South Africa. We aim to keep track of DNA barcoding in the country and to advise on areas for improvement and expansion to implement a more holistic and widely useful barcoding reference library. South Africa has an active research community that adopted DNA barcoding almost immediately after it was introduced, or perhaps even before [93], and that has been growing since then. Consequently, there were many private records (441,619; Table 1 and S2 Fig), which were comparable in quantity to those that have been made publicly available. Therefore, it is crucial to motivate the community to publish their data. Funders or academic institutions could make it a requirement that all sequencing data be submitted to genomic databases such as BOLD and GenBank, for accessioning, before theses or publications are submitted. This will not only help to improve the reference library but also reduce the amount of duplication that inevitably leads to a wasteful use of resources.

A consensus on DNA barcoding standards for plants to which BOLD can assign BINs need to be reached. This will need to be achieved through discussions within the plant research community and BOLD. Plants not assigned to BINs face exclusion from an efficient and effective curation system, and so it is critical to find a solution to this issue.

## Conclusion

DNA barcoding in South Africa has advanced significantly over the past decade, with expanded coverage across additional taxonomic groups, increased sampling from new localities, and improvements to the reference library. Nevertheless, the findings highlight major gaps that remain to be addressed. In particular, the study emphasizes the need for the South African DNA barcoding community to identify taxonomic groups that should be prioritized to enhance and expand the existing reference library. This includes facilitating discussions to make private BOLD records public, to inform a more comprehensive assessment of spatial and taxonomic gaps. Furthermore, private BOLD data not published on GenBank must be verified for accuracy before use. As more data become publicly available, SABOL can tap into the BOLD tools, such as hit lists and progress reports, to enable real-time tracking to monitor barcoding progress against the South African checklists of known species, including geographical representation. This approach will establish clear targets for both the research and policy community, while fostering collaboration, reduce duplication, and provide a platform for wider public engagement with the DNA barcoding community in South Africa.

## Supporting information

S1 Fig The number of South African records available on BOLD Systems contributed by the Centre for Biodiversity Genomics of the University of Guelph (Canada) versus the records submitted by South African institutions over time (categorised in four-year periods). (TIF)

S2 Fig The comparison of public and private South African BOLD records. Total number of South African records on BOLD broken down by public and private records.
(TIF)

S3 Fig The comparison of records with GenBank accession numbers and those without. Proportion of barcode records containing GenBank accession numbers compared to those without, across taxonomic groups.
(TIF)

S4 Fig BIN to species ratio regression analysis. The relationship between the number of BINs assigned to records and the number of species morphologically identified and assigned to species name, with the orange line indicating the 1:1 ratio (BIN to Species).
(TIF)

S1 Table Plant checklist. (XLSX)

S2 Table Fungi checklist. (XLSX)

S3 Table Alien checklist. (XLSX)

S4 Table File-appendix1-publications data. The downloaded publications that generated DNA barcodes recognised by iBOL or used at least one barcode marker to improve or undertake identifications.
(XLSX)

S5 Table Most used gene markers on GenBank. The three most used gene markers per taxonomic group from GenBank.
(XLSX)

S6 Table List of taxa missing in each province. A list of taxonomic groups that lack representation by at least one species in some South African provinces.
(XLSX)

S1 File Genetic data downloaded from GenBank for each taxon group. The data was downloaded in a text format and transferred to Microsoft excel for analysis.
(ZIP)
