# Supplementary figures and images for: An overview of DNA barcoding of biodiversity in South Africa
Source: PLoS One. 2026 Apr 21;21(4):e0345173. doi: 10.1371/journal.pone.0345173 (PMC13098986; doi:10.1371/journal.pone.0345173)

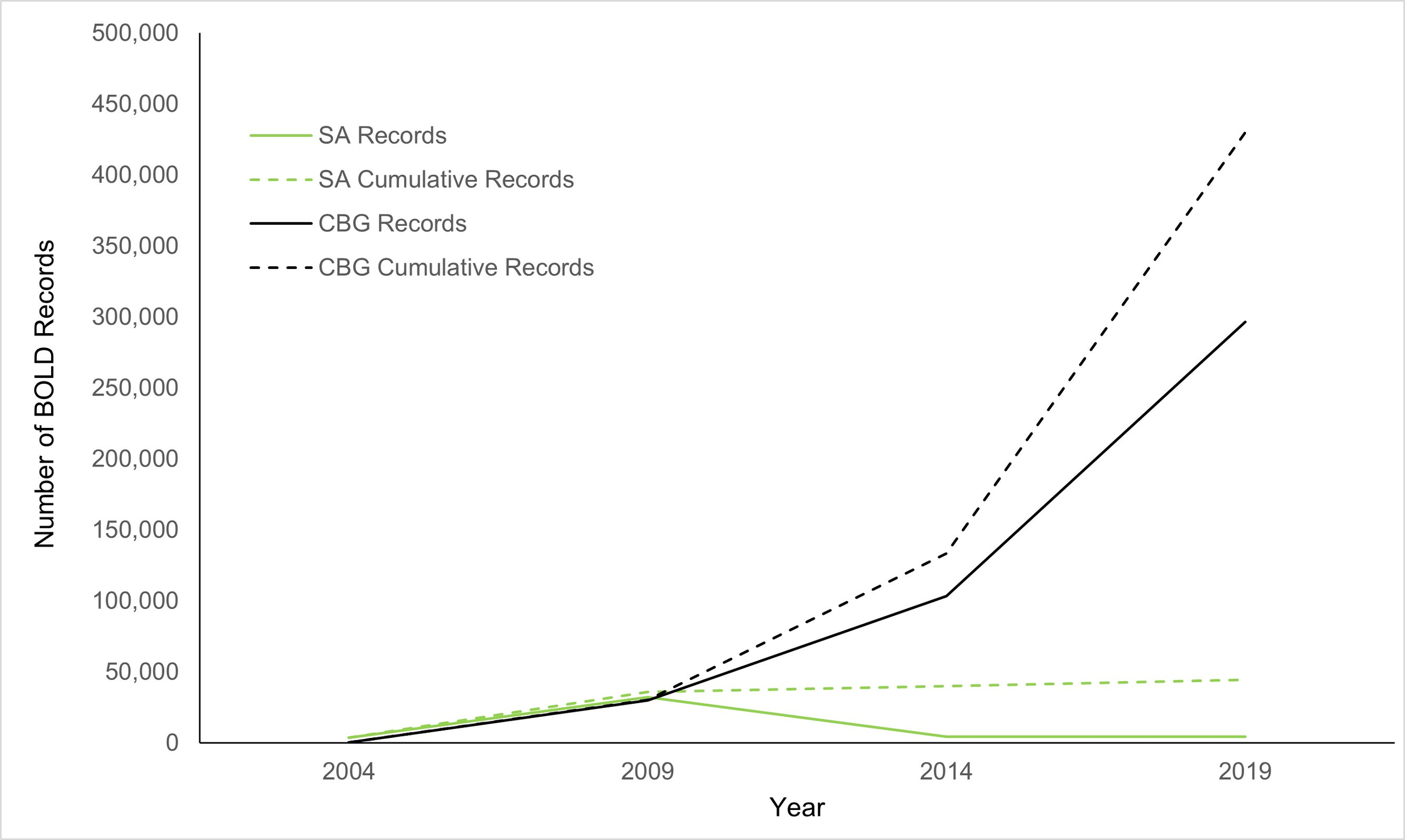

Supplement: S1 Fig — (TIF) [file pone.0345173.s001.tif]

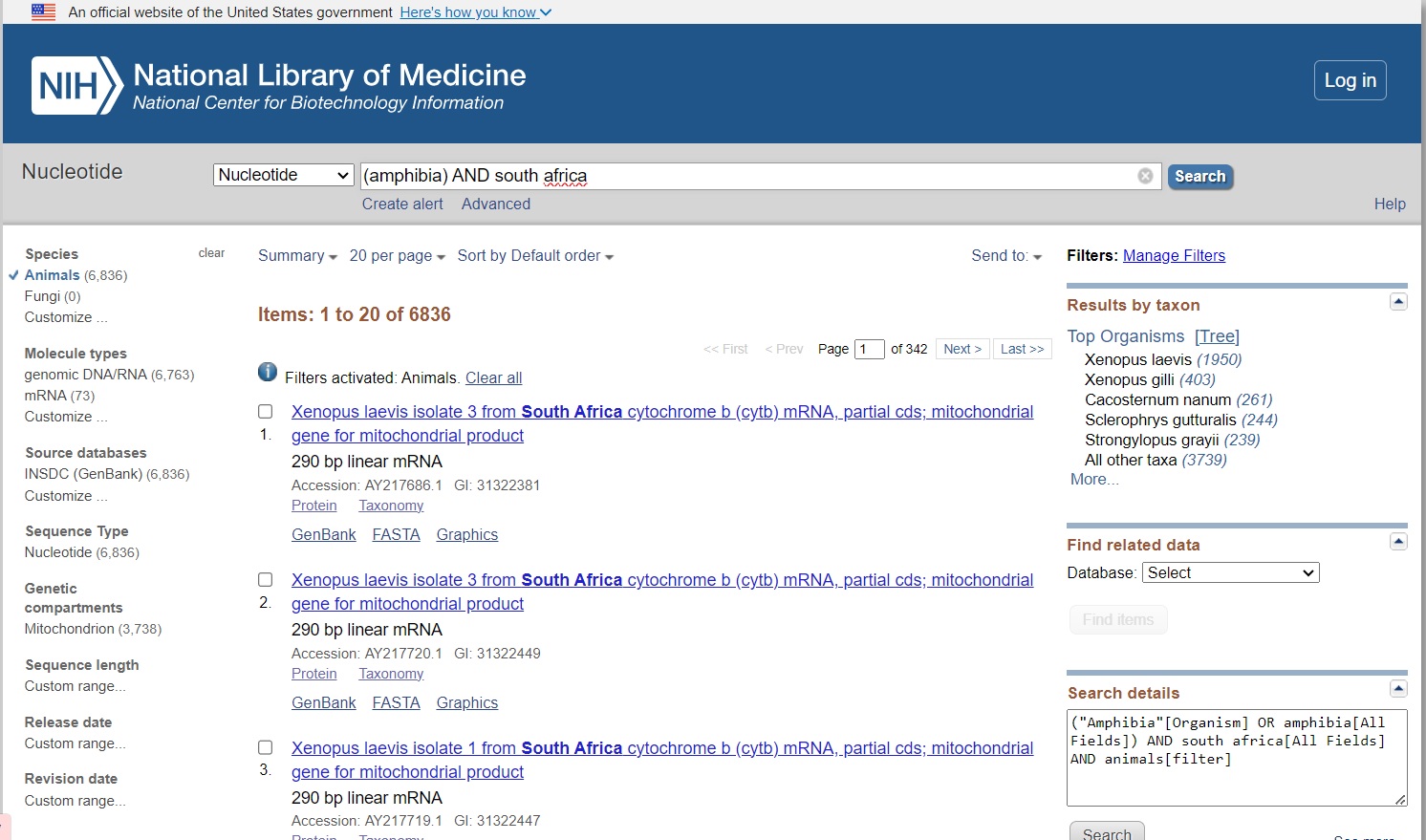

Supplement: S1 File — The data was downloaded in a text format and transferred to Microsoft excel for analysis. (ZIP) [file pone.0345173.s011.zip › S1_File/Amphibians.jpg]

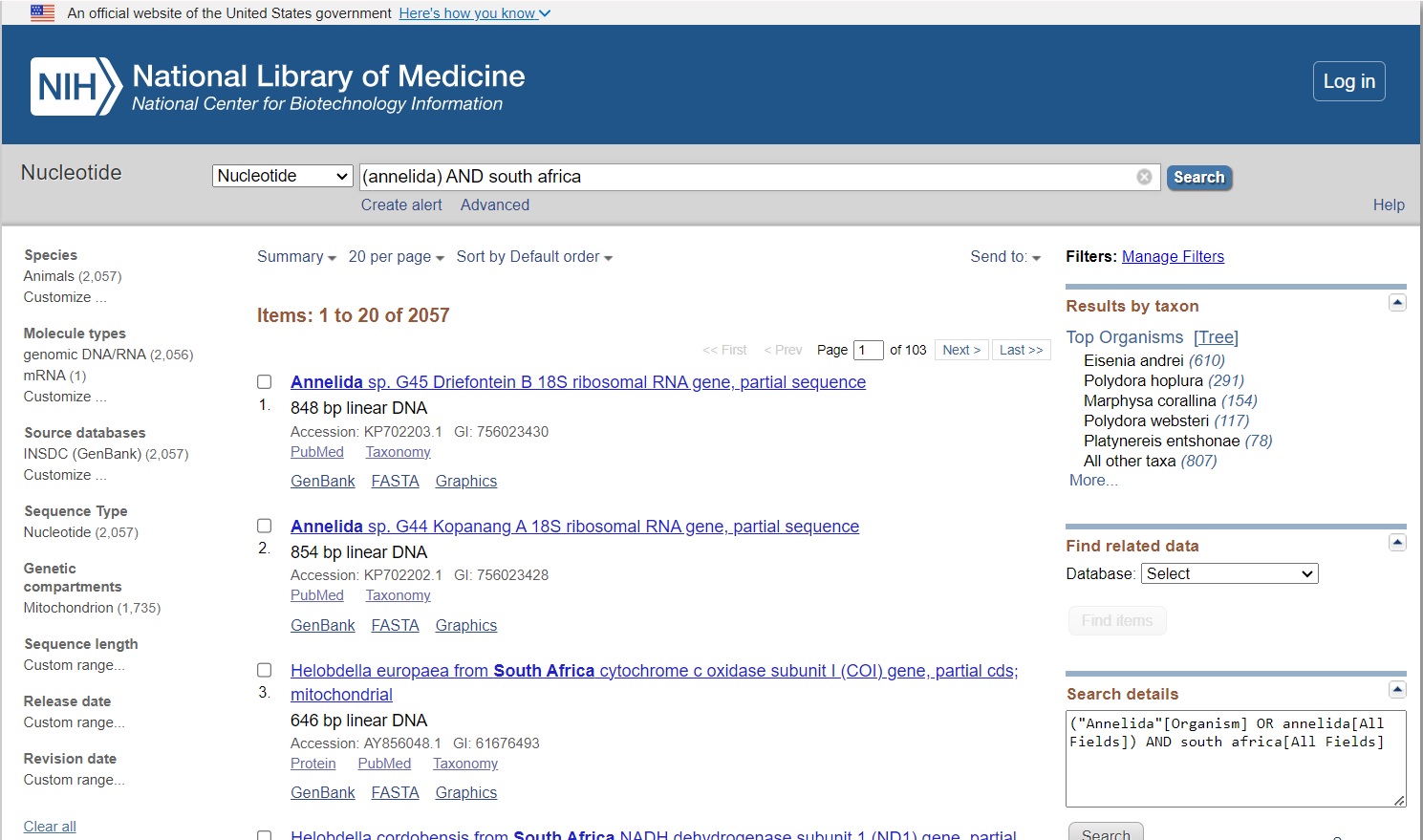

Supplement: S1 File — The data was downloaded in a text format and transferred to Microsoft excel for analysis. (ZIP) [file pone.0345173.s011.zip › S1_File/Annelids.jpg]

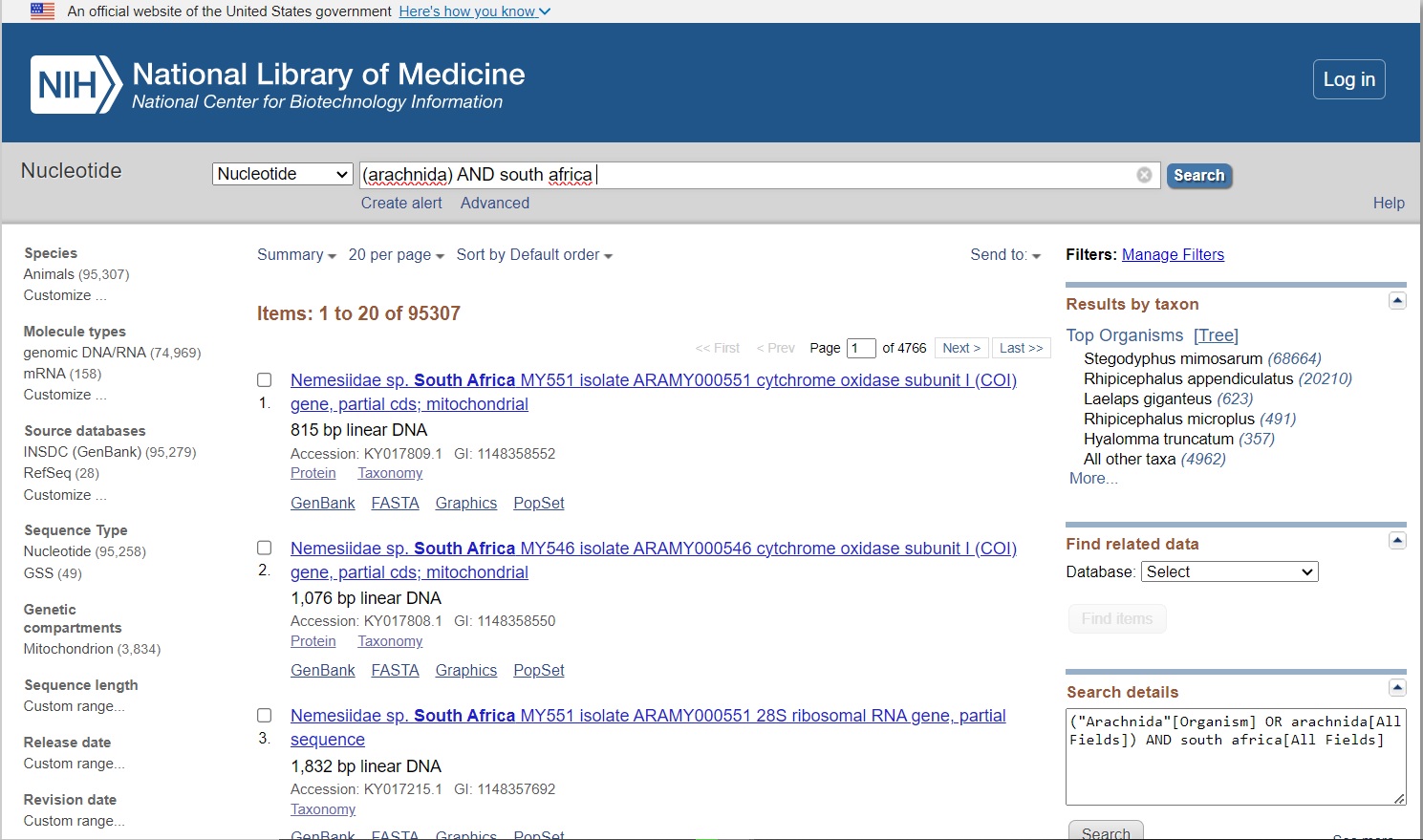

Supplement: S1 File — The data was downloaded in a text format and transferred to Microsoft excel for analysis. (ZIP) [file pone.0345173.s011.zip › S1_File/Arachnids.jpg]

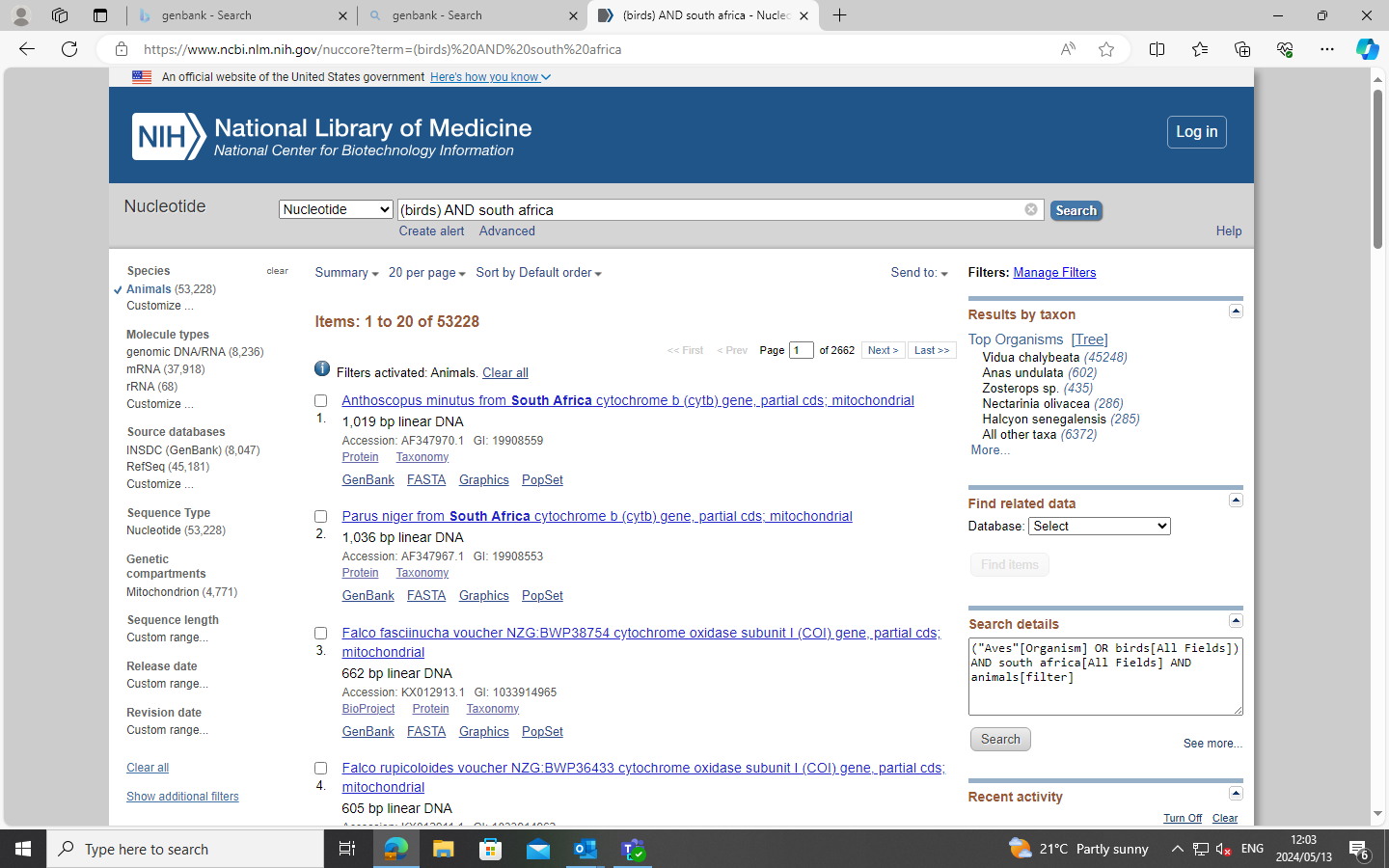

Supplement: S1 File — The data was downloaded in a text format and transferred to Microsoft excel for analysis. (ZIP) [file pone.0345173.s011.zip › S1_File/Birds.png]

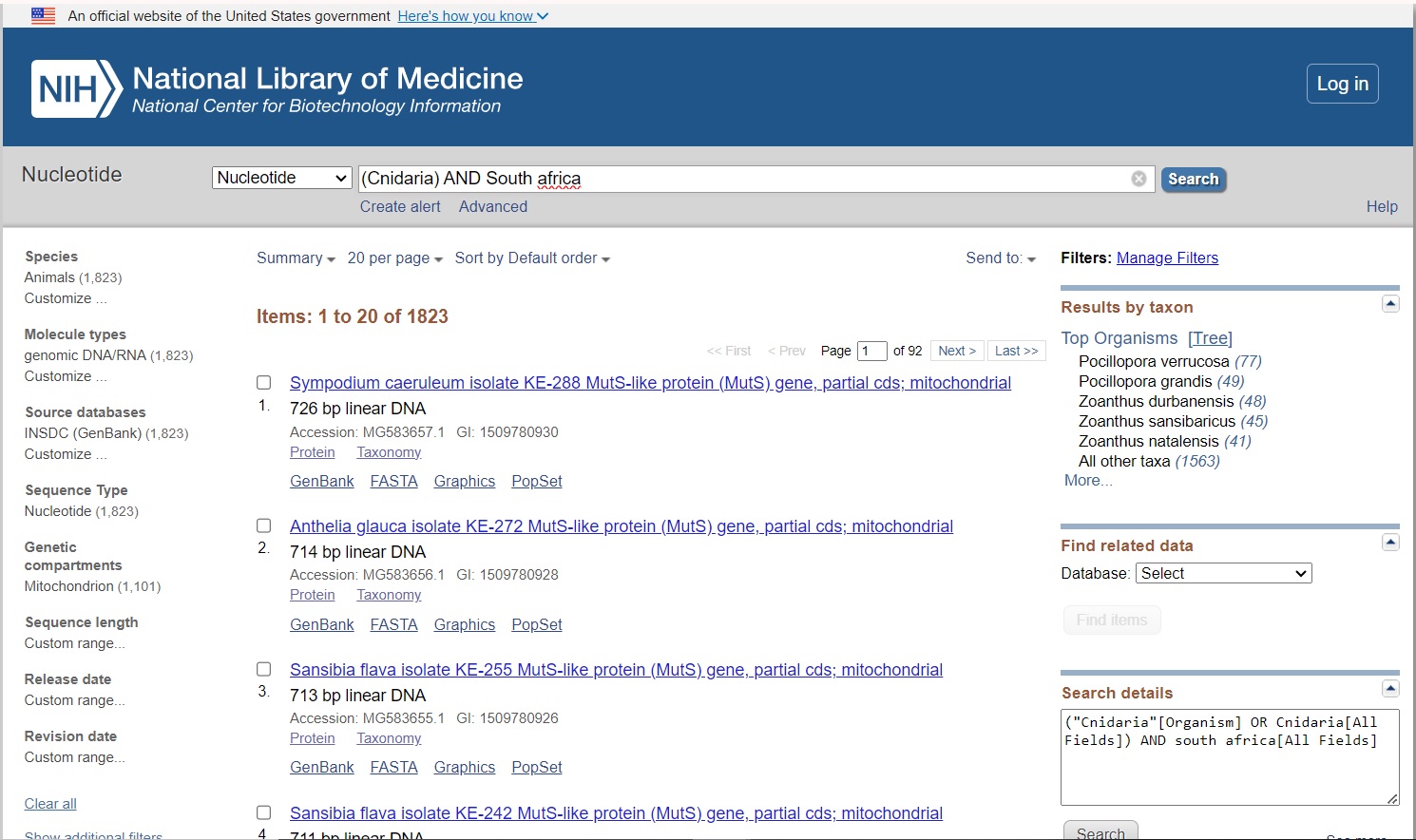

Supplement: S1 File — The data was downloaded in a text format and transferred to Microsoft excel for analysis. (ZIP) [file pone.0345173.s011.zip › S1_File/Cnidarians.jpg]

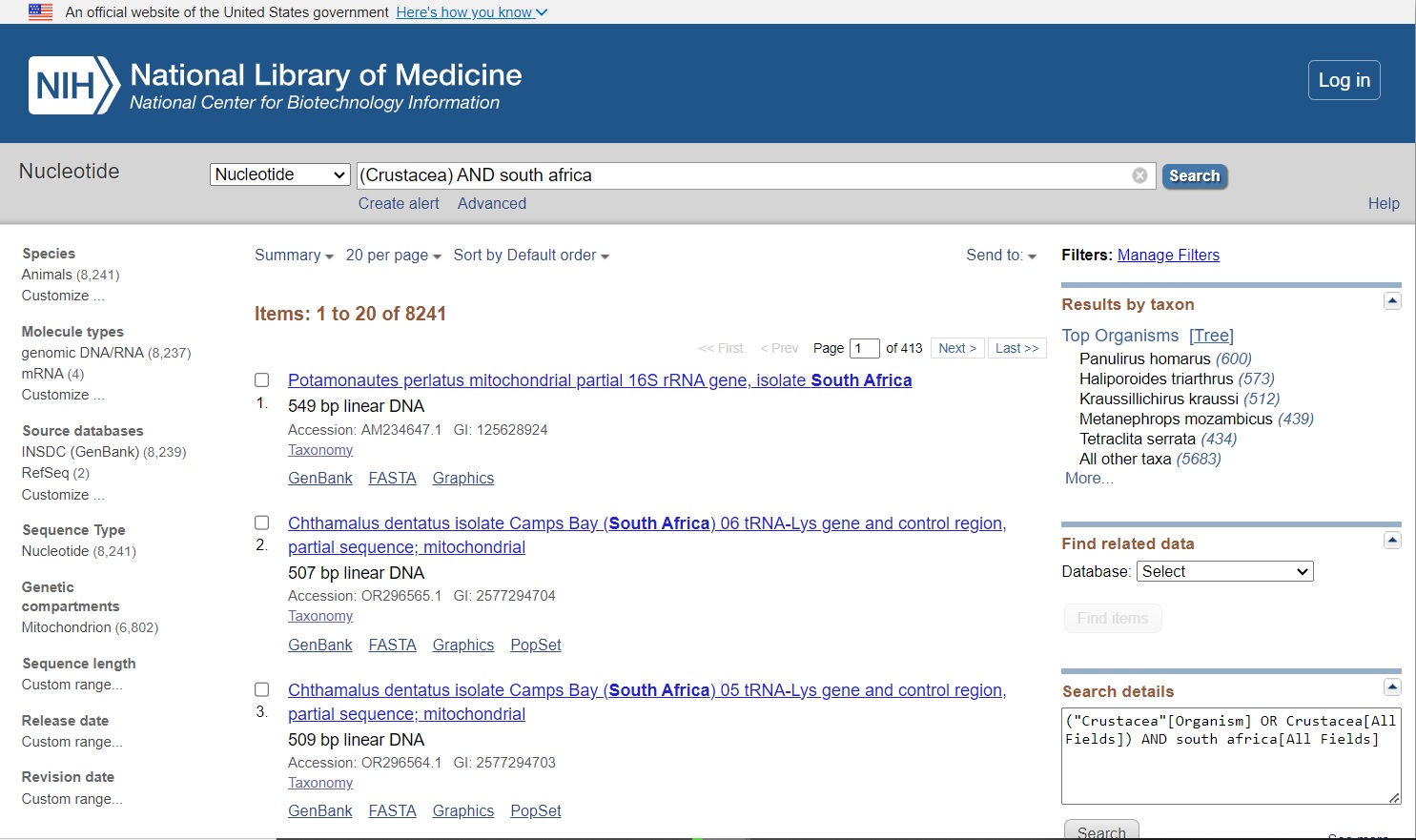

Supplement: S1 File — The data was downloaded in a text format and transferred to Microsoft excel for analysis. (ZIP) [file pone.0345173.s011.zip › S1_File/Crustaceans.jpg]

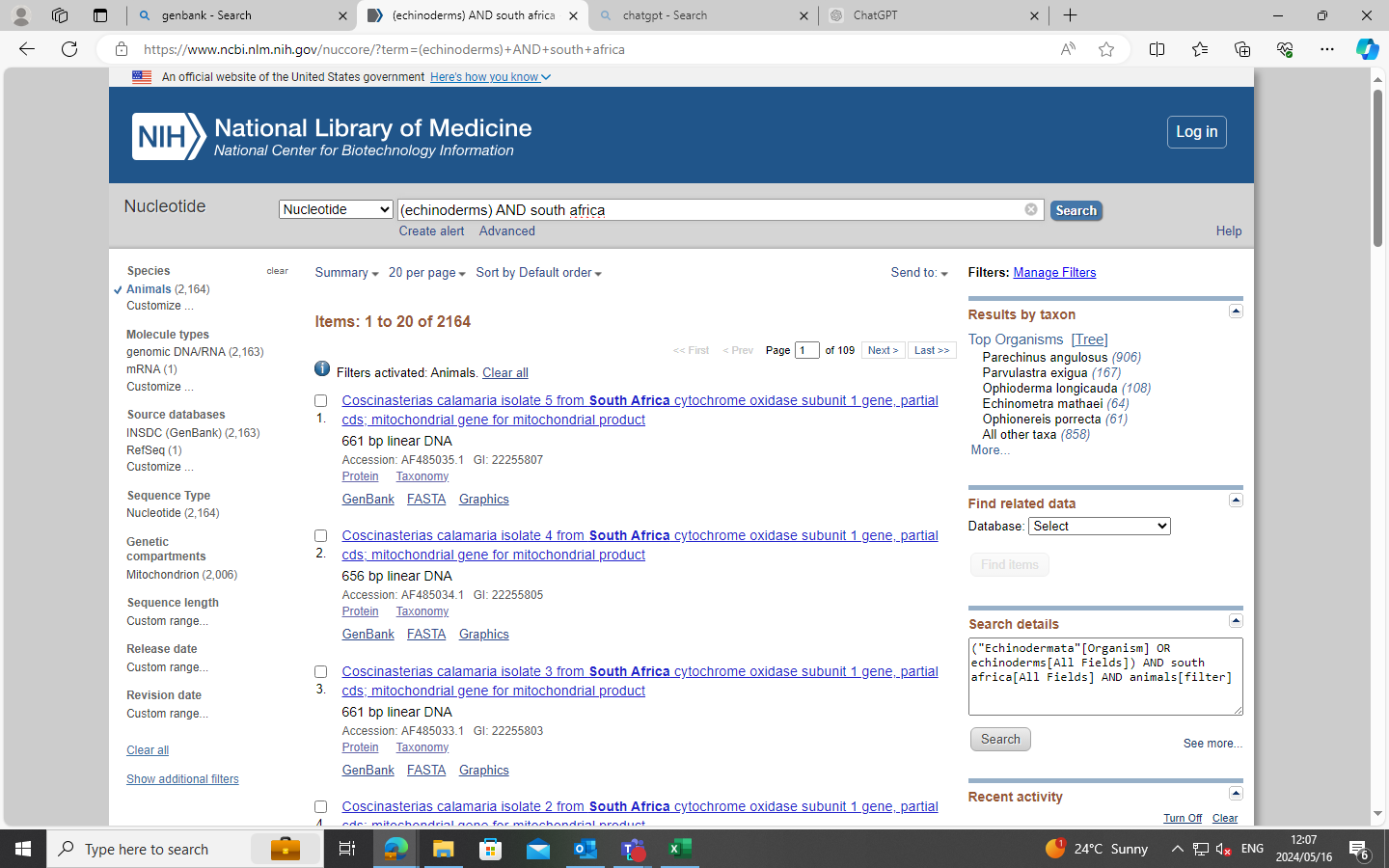

Supplement: S1 File — The data was downloaded in a text format and transferred to Microsoft excel for analysis. (ZIP) [file pone.0345173.s011.zip › S1_File/Echinoderms.png]

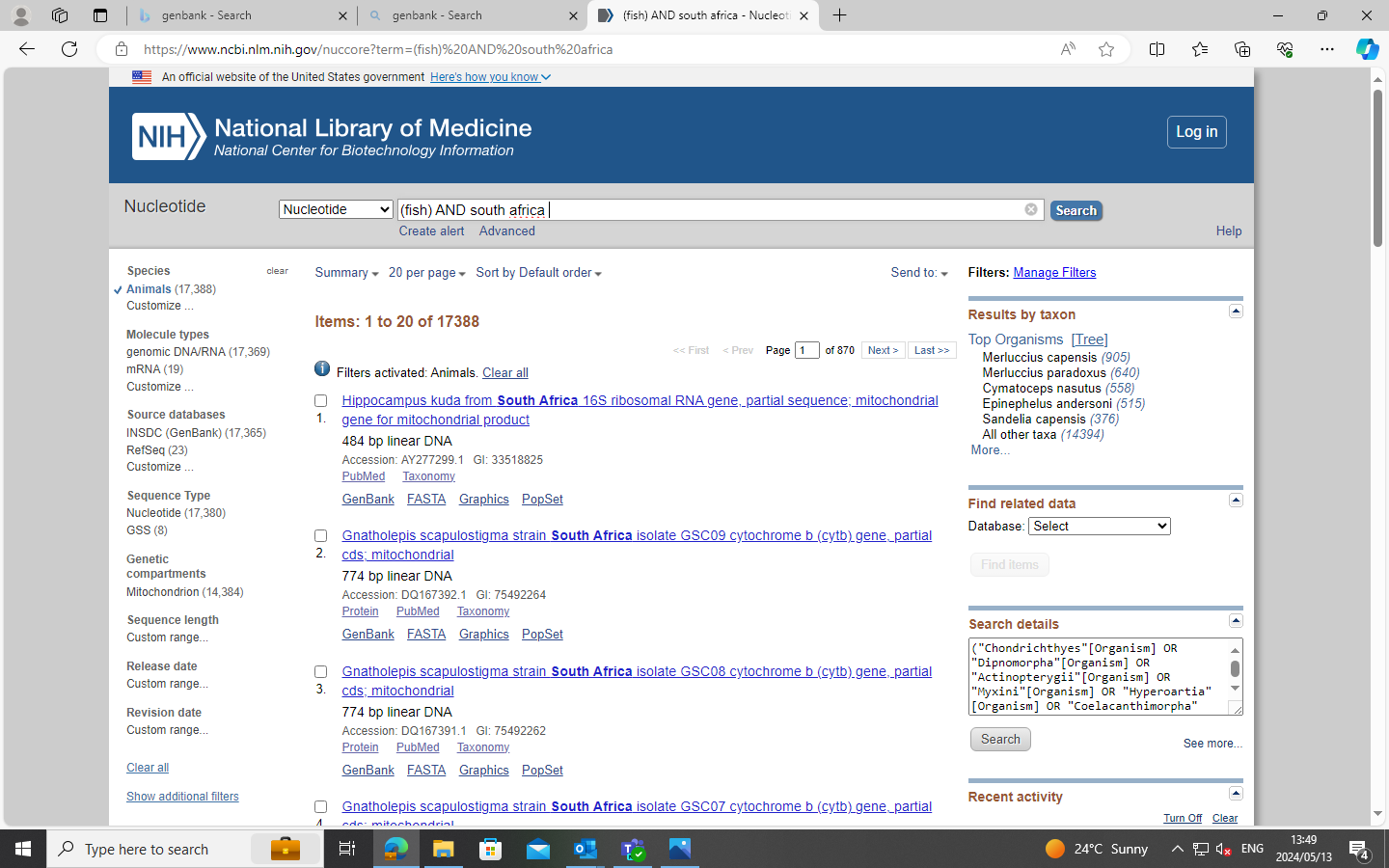

Supplement: S1 File — The data was downloaded in a text format and transferred to Microsoft excel for analysis. (ZIP) [file pone.0345173.s011.zip › S1_File/Fish.png]

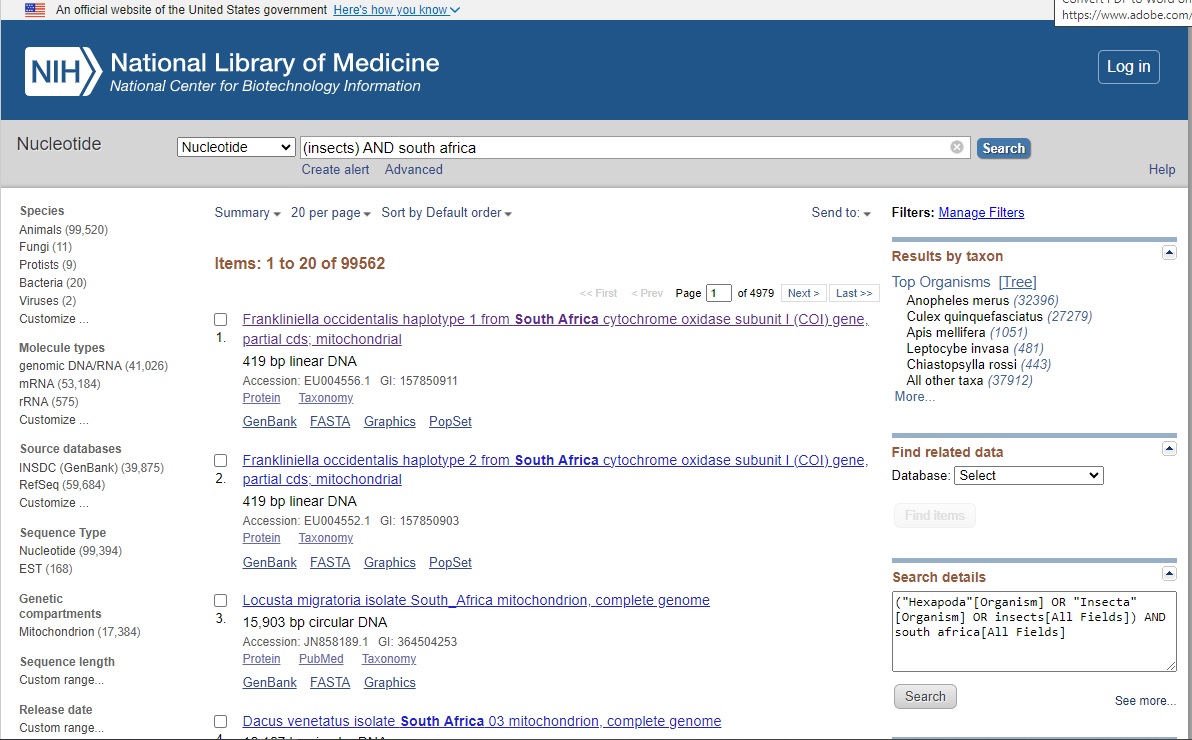

Supplement: S1 File — The data was downloaded in a text format and transferred to Microsoft excel for analysis. (ZIP) [file pone.0345173.s011.zip › S1_File/Insects.jpg]

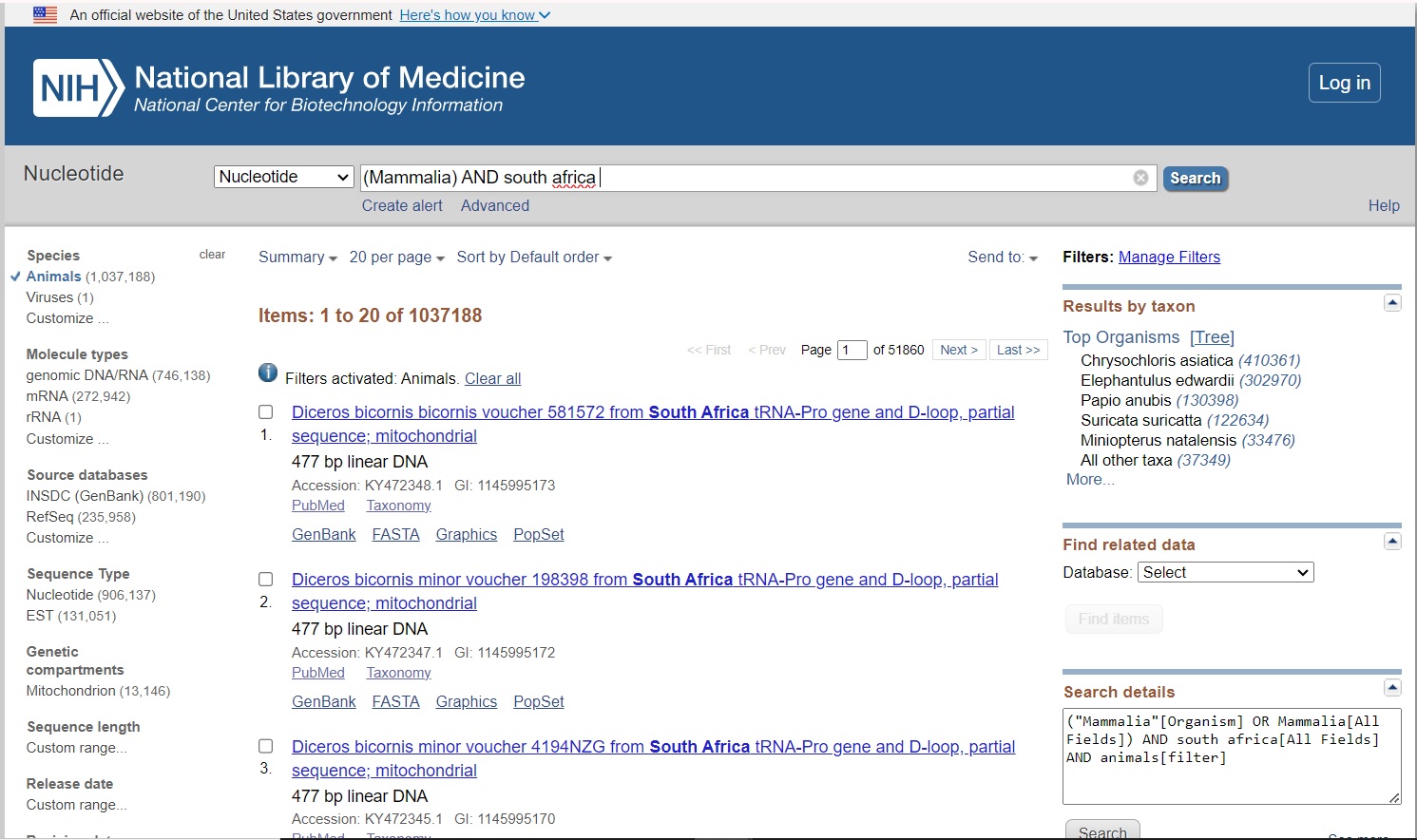

Supplement: S1 File — The data was downloaded in a text format and transferred to Microsoft excel for analysis. (ZIP) [file pone.0345173.s011.zip › S1_File/Mammals.jpg]

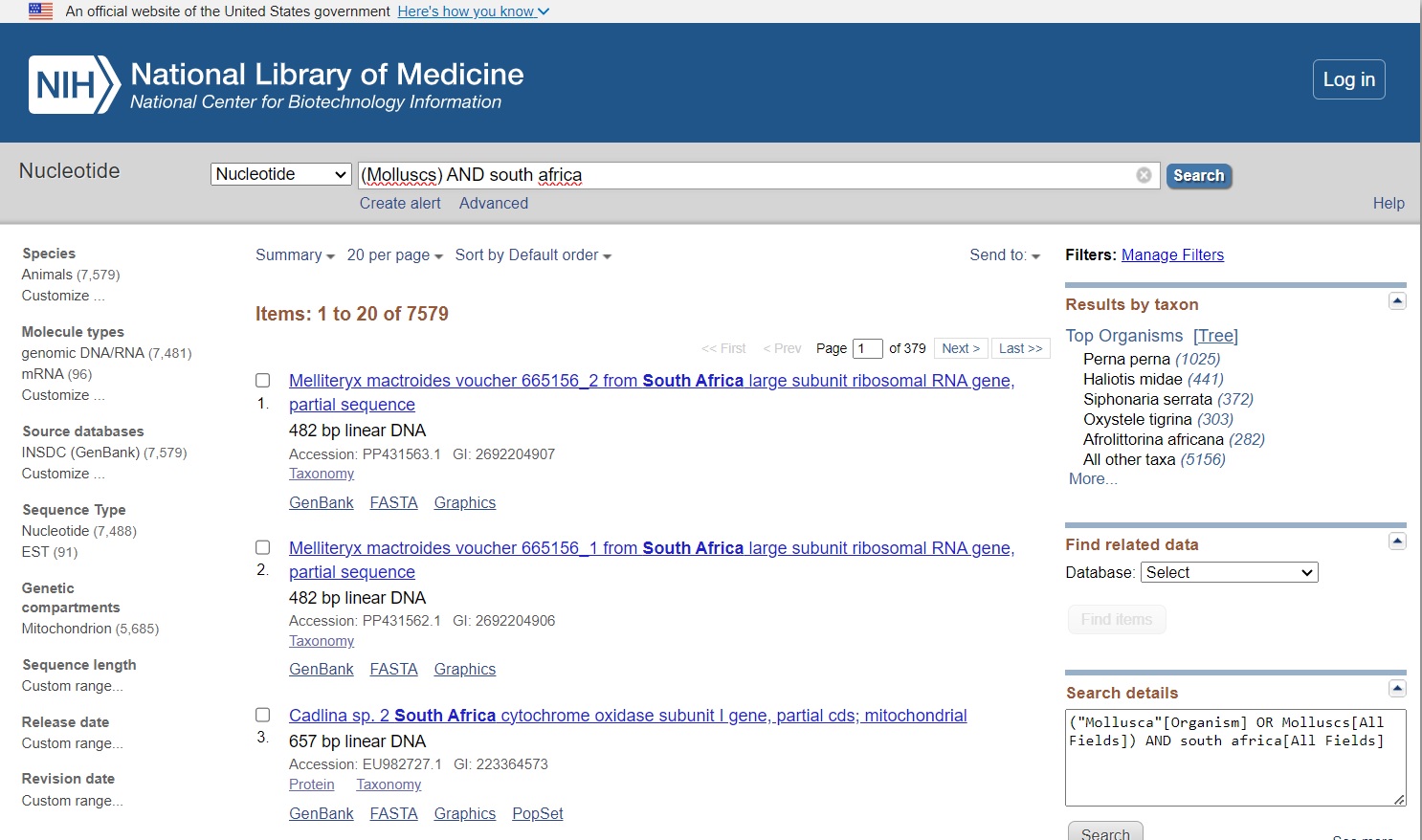

Supplement: S1 File — The data was downloaded in a text format and transferred to Microsoft excel for analysis. (ZIP) [file pone.0345173.s011.zip › S1_File/Molluscs.jpg]

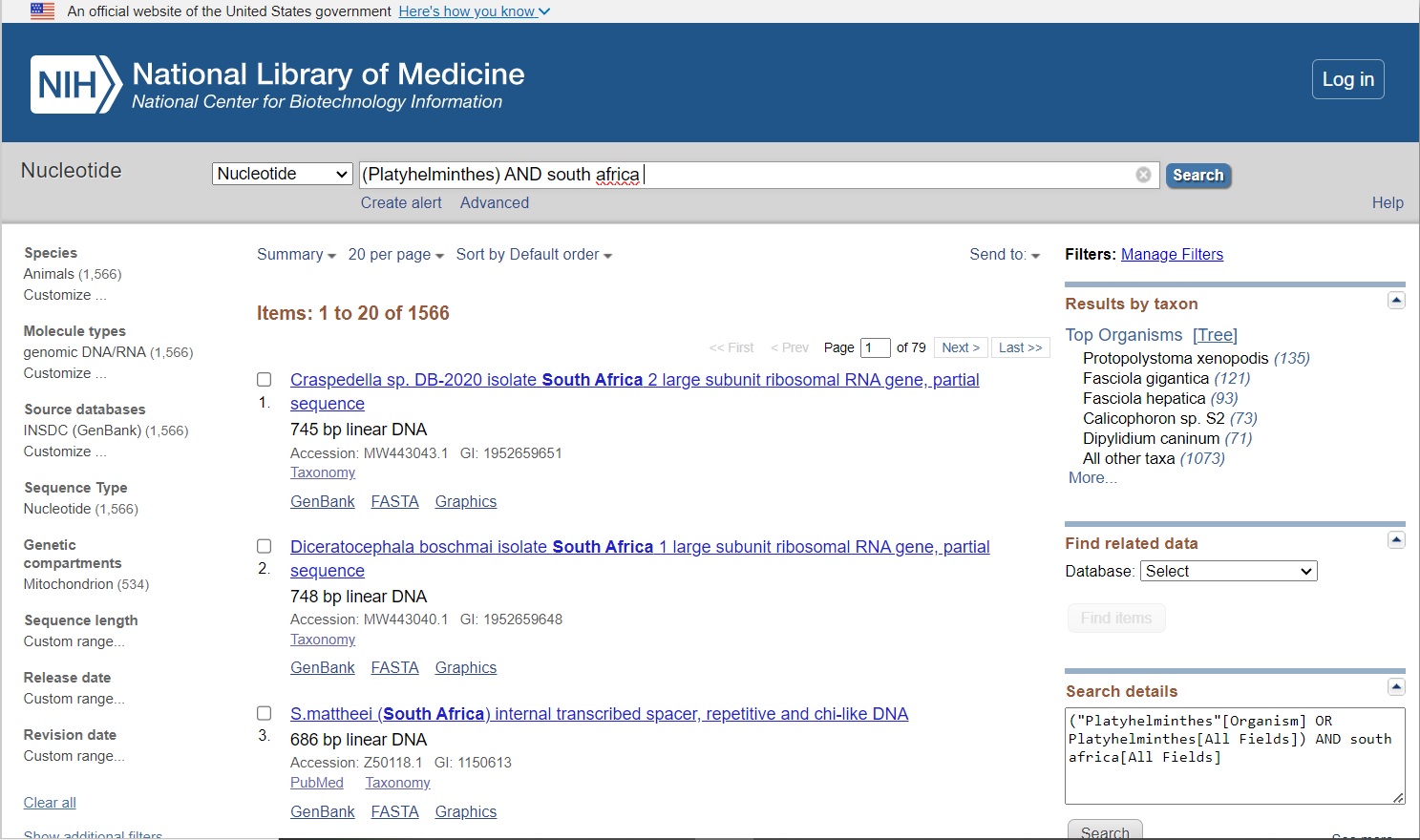

Supplement: S1 File — The data was downloaded in a text format and transferred to Microsoft excel for analysis. (ZIP) [file pone.0345173.s011.zip › S1_File/Platyhelminthes.jpg]

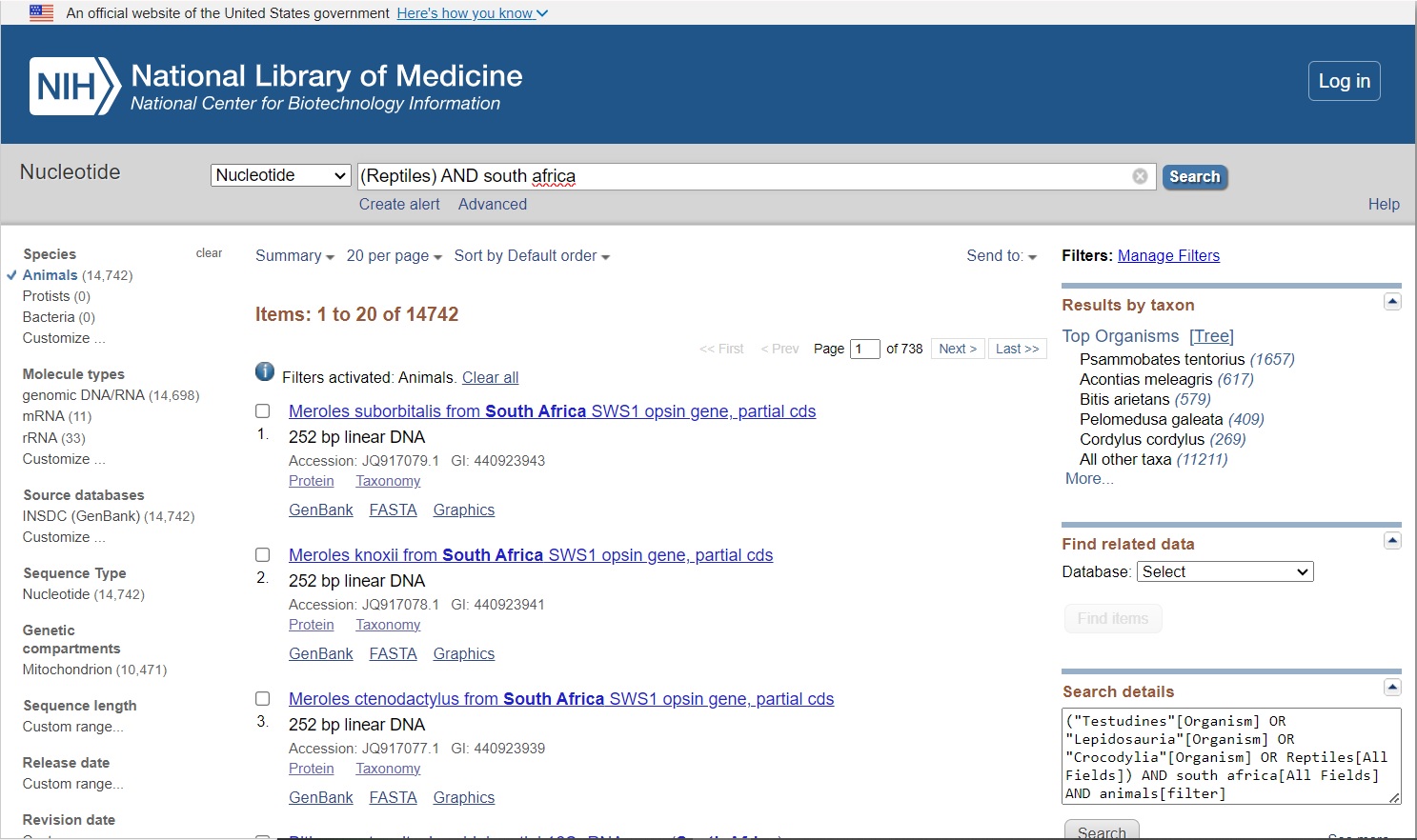

Supplement: S1 File — The data was downloaded in a text format and transferred to Microsoft excel for analysis. (ZIP) [file pone.0345173.s011.zip › S1_File/Reptiles.jpg]

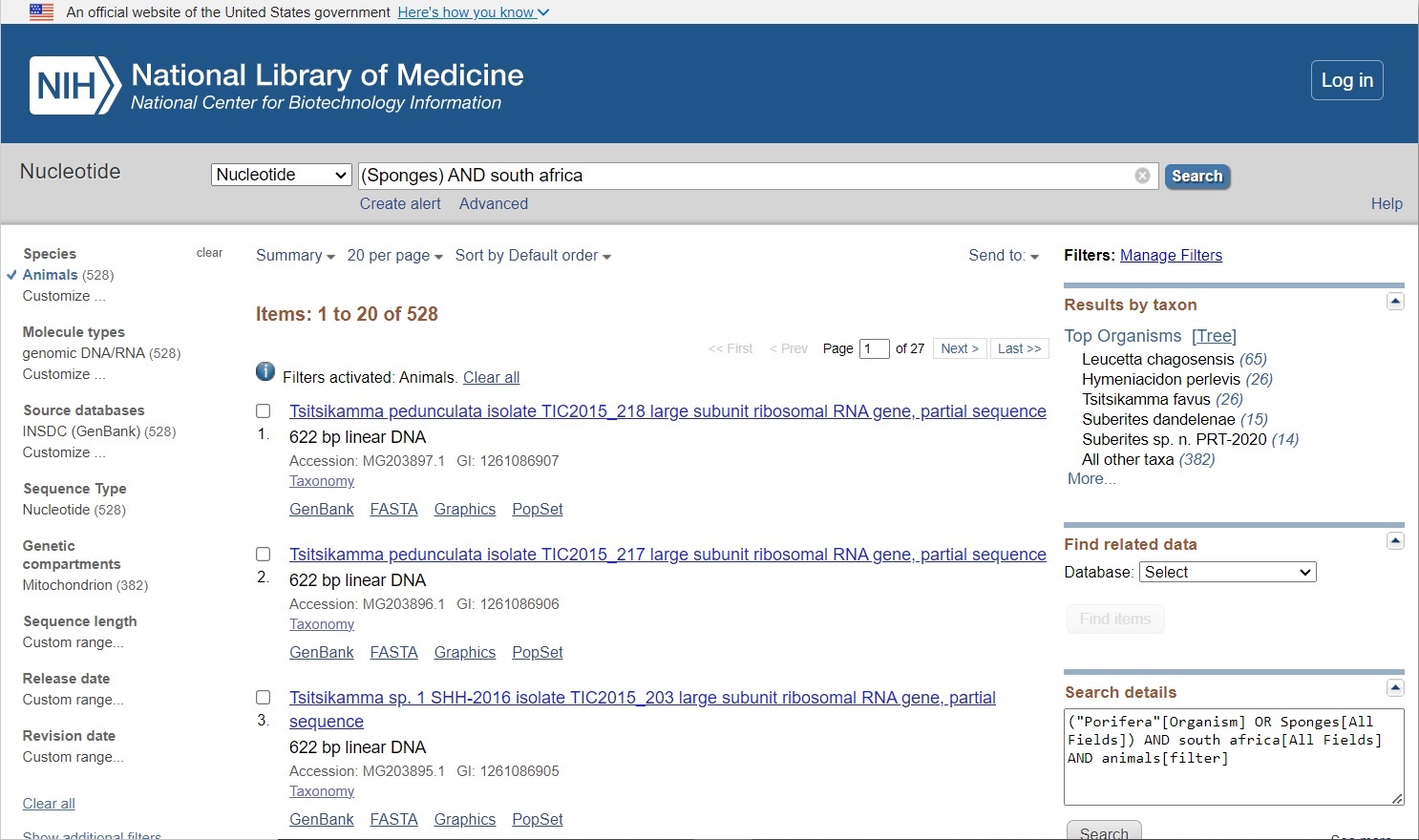

Supplement: S1 File — The data was downloaded in a text format and transferred to Microsoft excel for analysis. (ZIP) [file pone.0345173.s011.zip › S1_File/Sponges.jpg]
