# Supplementary material for: An overview of DNA barcoding of biodiversity in South Africa
Source: PLoS One. 2026 Apr 21;21(4):e0345173. doi: 10.1371/journal.pone.0345173 (PMC13098986; doi:10.1371/journal.pone.0345173)
Supplement: Attachment — Submitted filename: Comments to the authors_Plos One.pdf [file pone.0345173.s017.pdf]

## Comments to the authors

This study assesses the current status and progress of DNA barcoding in South Africa through a gap analysis, comparing verified species checklists with barcoded sequences from the Barcode of Life Database (BOLD) and GenBank to evaluate taxonomic and geographic representation. Some comments are provided for the authors' consideration to improve the quality and clarity of the manuscript.

## Abstract

Line 133- "BINS" should be corrected to "BINs."

Line 137- The standard usage of "cytochrome oxidase subunit I (COI)" is "cytochrome c oxidase subunit I (COI)."

Lines 138- The phrase "Discrepancies between GenBank records and the records mined by BOLD from GenBank…" repeats "records" and "GenBank," which reduces clarity. Consider rephrasing to make it more concise and readable.

Lines 142- The phrase "resolve BIN assignment for inconsistent taxonomic assignments" is unclear. Rewriting it as "resolve inconsistencies in BIN assignments, particularly for underrepresented groups" would improve clarity and precision.

## Introduction

The Introduction is well-researched, authoritative, and highly relevant to the topic, but it would benefit from greater concision. The section is overly long and at times diffuse, particularly in lines 167-213 where extensive background on taxonomy history, the Global Taxonomy Initiative, species concepts, and ICZN recommendations may exceed what is necessary to frame a review of DNA barcoding progress in South Africa. For citing in the text follow the journal guideline, such as Hebert et al. (2003) not Hebert et al. [12] line 505.

Several points would benefit from clarification or refinement-

Line 156- The reference to "three of the 35 globally recognized biodiversity hotspots" lacks specificity. Please name the three hotspots explicitly to improve clarity and regional relevance.

Line 157- Percentages and absolute species numbers are used interchangeably, which may reduce interpretability. Consider using one consistently, or explicitly justify the use of both.

Line 170- less than 14% of global species diversity has been documented is dependent on uncertain estimates. This claim should be qualified using cautious language (e.g. "estimated" or "likely").

Line 187- While standard barcode regions are listed, their known limitations are not mentioned. Briefly noting constraints-such as lower discriminatory power in plants and fungi-would provide a more balanced overview.

Line 193- The controversy surrounding DNA barcoding is acknowledged but not explained. Adding a brief clause summarising key criticisms (e.g. reliance on single-locus data) would strengthen the discussion.

Line 206- The sentence is overly long and grammatically awkward. Splitting it into two sentences would improve clarity.

Line 216- “Continents” should be  revise to “continents.”

Line 248- Objectives (1) and (2) partially overlap. Refining them to ensure each addresses a distinct analytical component would improve conceptual clarity.

**Materials and Methods**

The Materials and Methods section is thorough and generally well aligned with the study objectives. However, greater methodological transparency, clearer operational definitions, and explicit justification of analytical choices are needed to enhance reproducibility and reviewer confidence, particularly given the data-intensive nature of the review.

Specific points requiring attention include-

Line 259- The SANBI national plant checklist is cited, but the date of data download is not provided. Please add the exact download date, as is done for other datasets.

Line 262- Multiple fungal data sources are listed, but the strategy for integrating them is unclear. Clarify whether sources were merged, prioritised, or cross-validated, and explain how conflicting names or classifications were resolved.

Line 264- The phrase “Index Fungorum () database” contains empty parentheses. These should be removed or completed.

Line 266- Using a single search term (“fungi South Africa”) is overly simplistic and likely incomplete, risking omission of taxa due to synonymy, spelling variation, or missing locality metadata.

Line 268- Animal checklists are attributed to “taxon experts,” but criteria for expert selection and validation are not specified. Indicate whether these lists were peer-reviewed or cross-checked against recognised taxonomic authorities.

Line 273- Treating records containing qualifiers such as “cf.”, “aff.”, or “nov.” as valid species names is problematic and may inflate species counts, biasing gap analyses.

Lines 276-278- The statement that “problematic records were individually checked” lacks quantification. Please indicate how many records required manual correction.

Line 282- Restricting searches to records labelled “South Africa” in BOLD may miss entries with incomplete or erroneous geographic metadata. This limitation should be acknowledged.

Line 294- The treatment of uncertain species identifications as valid species is reiterated. To avoid redundancy, refer back to the justification provided earlier (line 273).

Line 301- Key terms such as “distinct records” and “iBOL-compliant” are not operationally defined and should be clearly specified.

Line 303- Barcode Index Numbers (BINs) are COI-specific. Counting BINs across barcode regions may therefore be conceptually misleading. Clarify explicitly that BIN-based analyses apply only to animal COI records.

Line 306- BIN splits and merges are interpreted as indicators of taxonomic issues without consideration of misidentification. Please clarify whether records were screened for misidentifications.

Line 316- Analyses are restricted to records assigned BINs, excluding potentially informative non-BIN barcode data. Justify this exclusion and discuss its implications, particularly for plant and fungal datasets.

Line 328- The study is not described as a systematic or scoping review. Please justify the exclusion of publications prior to 2004 and clarify whether earlier relevant studies exist.

Line 333- The term “[molecular term]” appears in a third brackets without explanation. Please clarify its purpose or remove it.

Line 349- The comparison between GenBank and BOLD records lacks methodological detail. Specify how overlap was assessed (e.g. accession numbers, species names, markers) and indicate whether discrepancies were quantified or only qualitatively described.

**Results**

The Results section provides a comprehensive and data-rich quantitative assessment of South African biodiversity representation on BOLD. The statistics on species counts, public versus private records, BIN assignments, and taxon-specific barcoding coverage are informative and effectively highlight major representation gaps. However, improvements can be made in clarity, consistency, and precision. Some sentences are overly long or contain dense combinations of percentages and ratios that may hinder readability. In addition, certain methodological assumptions such as treating each BIN as a distinct species lineage would benefit from clearer justification or explicit acknowledgement of their limitations.

Specific points requiring attention include-

Lines 356-358- The presentation of species counts and percentages is clear, but consistency would improve readability. Consider using either absolute numbers or percentages uniformly, or presenting both in a parallel structure. For example-

“Animals are the most represented group with 58,182 species (63%), followed by plants with 21,524 species (23%) and fungi with 12,581 species (14%).”

Lines 359-361- The distinction between public and private records is clearly described. However, the estimate of approximately 13 million global records would benefit from a citation or a brief note indicating when this figure was retrieved, as database content changes over time.

Lines 362-366- The rationale for analysing only public records is appropriate but could be stated more succinctly. For example-

"Only publicly accessible records were analysed, as private records could not be accessed."

Lines 372-374- The explanation for the absence of BIN assignments for certain unique names is reasonable. Consider a slight rephrasing for clarity-

"The 3,832 unique names not assigned to BINs likely reflect non-compliance with iBOL barcode standards or missing sequence data for the corresponding specimens."

Lines 382-384- Fungi and plants are identified as underrepresented groups. For plants, the text notes that the two-gene barcode system (matK and rbcLa) could not be fully captured. It would strengthen the Results section to briefly explain why this limitation exists (e.g., BOLD platform constraints, incomplete marker coverage, or data formatting issues).

Table 1- The absence of BINs for fungi is a notable result and should be more clearly explained in the Results text. Explicitly state that BINs are based on COI sequences and are therefore not applicable to fungal ITS barcodes (this could also be reinforced with a table footnote).

Similarly, the presence of only 119 BINs for 3,469 plant species requires clarification. Briefly explain in the Results that BINs are largely incompatible with standard plant barcoding markers, which accounts for the low BIN representation despite relatively high species counts.

**Discussion**

The discussion is comprehensive, data-rich, and well-referenced, providing a detailed account of South Africa's progress in DNA barcoding, its applications, limitations, and future directions. The section demonstrates a strong command of the literature and presents the material in a largely logical order, moving from progress and data trends - reference library gaps - applications - challenges - recommendations and future directions.

However, the discussion could benefit from improved clarity, conciseness, and integration of key findings. At times, the section is dense, with long paragraphs and repeated points, which may reduce readability for non-specialists. Some minor inconsistencies, e.g., the use of percentages versus absolute numbers or redundant explanations of BIN/OTU, could be streamlined.

Paragraphs are very long (e.g., lines 502-554, 734-762), often covering multiple points. Suggest breaking long paragraphs into smaller, thematic paragraphs (e.g., separate methods/technical challenges, applications, capacity-building, and funding).

Data presentation is sometimes confusing, mixing percentages and absolute numbers. Example- lines 518-520- "animal records have increased by 890%, insects 973%"-readers may benefit from absolute numbers for context.

Line 553- OUT should be OTU

Line 625- COI is used for plants; previously, it was clarified that matK and rbcLa are standard. Consider briefly clarifying the historical context versus current standards.

Lines 858-860- Discussion of plant BINs is a bit unclear; needs stronger linkage between issue (COI misassignment) and recommended solution.

The discussion refers to multiple figures and tables (S3, S5, S8, S11, Fig 2, Fig 3, Fig 6), but these could be better integrated into the narrative with key takeaways highlighted.

Consider summarizing major figure points in the text rather than just referencing them.

Some sentences are dense and could be simplified without losing meaning. Examples- lines 805-811, lines 833-841. Shorter sentences would improve readability.

Clarify the COI limitation discussion- why COI is used, where it fails, and alternatives.

Ensure all acronyms (e.g., HTS, eDNA, SABOL, FBIP) are consistently defined at first use.

**Conclusion**

The conclusion would be stronger if it briefly reiterated one or two headline results (e.g., overall barcoding coverage, major taxonomic or geographic gaps) to anchor the recommendations in the study's core findings.

Consider reducing repetition of "should" by varying sentence structure.
