# Supplementary material for: An overview of DNA barcoding of biodiversity in South Africa
Source: PLoS One. 2026 Apr 21;21(4):e0345173. doi: 10.1371/journal.pone.0345173 (PMC13098986; doi:10.1371/journal.pone.0345173)
Supplement: Attachment — Submitted filename: Response to Reviewers.docx [file pone.0345173.s015.docx]

**Reviewer #1: This paper presents a gap analysis of the level of coverage of DNA barcoding for South African biodiversity by comparing taxonomic checklists with DNA barcode records in BOLD and GenBank. Below are some comments for the authors to consider to improve their manuscript:**

**Reviewer #1: Comment 1**

Page 24, Line 508 (now page 25-26, Line 538-541): Could it be that most of the records lacking family, genus, or species names originate from insects collected using Malaise traps? Malaise trap samples often contain many dark taxa—micro-insects that are challenging to identify morphologically (e.g., micromoths). Using BOLD for their identification is difficult due to the relatively incomplete reference library for African fauna. Please discuss this point.

**Authors comments:**

Yes, you are correct most of the taxa that have not been identified to the species level are insects. We have therefore updated the text in the paper for clarity as you have suggested (see line 538-541).

**Reviewer #1: Comment 2**

Visualizing Taxonomic Coverage: To better illustrate the level of coverage across taxonomic ranks, consider plotting a histogram. The x-axis could represent taxonomic ranks (order, family, genus, species), while the y-axis could show “representativity” (i.e., the percentage of taxa that have been barcoded at each rank). Additionally, for each rank, you could plot two bars: one representing the percentage of species barcoded and a second representing the percentage of species with more than five barcodes, which would provide insight into intraspecific sampling effort.

**Authors comments:**

Thank you for the comments. We have incorporated your suggestions and generated graphs to show the number of barcodes and taxonomic ranks, see graph below and Line 581-582 (citing S3 Fig) and Line 593 (citing S5 Fig).

**S3 Fig: The percentage of species records meeting DNA barcode standards for each taxon group**. The total number of species per taxon is given in brackets, divided into three categories: species with 1) zero barcodes, 2) one to four barcodes and 3) five or more barcodes.

**S5 Fig: The proportion of each taxonomic classification level per taxon group**. The number of records attributed to each taxonomic classification category within each taxon group. For each taxonomic group the total number of records represented is given in brackets.

**Reviewer #1: Comment 3**

Insect Barcoding Coverage: Since most BOLD records correspond to insects—especially Diptera due to global Malaise trap campaigns—it would be useful to zoom in on South African insect data to assess potential disparities in barcode coverage among different insect orders.

**Authors comments:**

We have updated the paper and added a more detailed table on South African insects, see table below and Line 536-538 and 541-544 (citing S11 Table).

**Table 11:** **Summary statistics of South African insects mined from BOLD as of 19 October 2023**. BINs - Barcode Index Numbers; BOLD - Barcode of Life Database Systems; SA - South Africa.

| Insect Taxa | Estimated No. of known species on SANBI Checklist | SA species in BOLD | % of SA species in BOLD | No. of BINs | BIN to species ratio | No. of BIN Merge/ total BIN merge | No. of BIN split/ total BIN split | Specimen without BIN no. | No. of Published records in BOLD |
| --- | --- | --- | --- | --- | --- | --- | --- | --- | --- |
| Archaeognatha | 18 | 0 | 0 | 1 | 0 | 0 | 0 | 129 | 130 |
| Blattodea | 251 | 11 | 4 | 152 | 13.8 | 0 | 2/4 | 415 | 1515 |
| Coleoptera | 6843 | 337 | 4.9 | 2993 | 8.9 | 3/7 | 26/61 | 9514 | 29910 |
| Dermaptera | 93 | 0 | 0 | 3 |  | 0 | 0 | 6 | 9 |
| Diptera | 6304 | 400 | 6.3 | 7602 | 19 | 24/58 | 45/119 | 26897 | 182229 |
| Embioptera | 10 | 1 | 10 | 31 | 31 | 0 | 1/2 | 40 | 139 |
| Ephemeroptera | 125 | 7 | 5.6 | 63 | 9 | 0 | 0 | 56 | 212 |
| Hemiptera | 1227 | 175 | 14.3 | 2327 | 13.3 | 1/2 | 11/23 | 11552 | 51087 |
| Hymenoptera | 6942 | 463 | 6.7 | 9277 | 20 | 16/35 | 46/129 | 23062 | 94857 |
| Lepidoptera | 11932 | 1129 | 9.5 | 4605 | 4 | 37/92 | 89/190 | 7168 | 41755 |
| Mantodea | 0 | 5 | 0 | 83 | 16.6 | 0 | 0 | 73 | 408 |
| Mecoptera | 31 | 0 | 0 | 2 | 0 | 0 | 0 | 1 | 22 |
| Megaloptera | 6 | 0 | 0 | 3 | 0 | 0 | 0 | 1 | 5 |
| Neuroptera | 459 | 48 | 10.5 | 162 | 3.3 | 0 | 2/4 | 374 | 2184 |
| Notoptera | 0 | 1 | 0 | 0 | 0 | 0 | 0 | 11 | 11 |
| Odonata | 273 | 29 | 10.6 | 38 | 1.3 | 2/4 | 1/2 | 104 | 246 |
| Orthoptera | 1361 | 39 | 2.9 | 340 | 8.7 | 2/4 | 8/16 | 1124 | 3797 |
| Phasmatodea | 0 | 1 | 0 | 3 | 1 | 0 | 0 | 21 | 24 |
| Plecoptera | 32 | 27 | 84.4 | 32 | 1.2 | 4/12 | 5/11 | 1 | 129 |
| Psocodea | 1 | 16 | 1600 | 180 | 11.3 | 0 | 3/6 | 1197 | 4221 |
| Siphonaptera | 104 | 3 | 2.9 | 5 | 1.7 | 0 | 0 | 3 | 36 |
| Strepsiptera | 10 | 1 | 10 | 7 | 7 | 0 | 0 | 7 | 31 |
| Thysanoptera | 293 | 19 | 6.5 | 260 | 13.7 | 0 | 3/11 | 798 | 3216 |
| Trichoptera | 169 | 42 | 24.9 | 101 | 2.4 | 1/2 | 5/12 | 41 | 822 |
| *Unidentified Records | 0 | 0 | 0 | 2 | 0 | 0 | 0 | 53 | 55 |
| Zygentoma | 0 | 0 | 0 | 8 | 0 | 0 | 0 | 5 | 19 |
| Total | **36484** | **2754** | **7.55** | **28280** | **10.3** | **90/216** | **247/590** | **82653** | **417069** |

*Records identified to phylum level, Insecta.

**Reviewer #1: Comment 4**

BIN-Taxonomy Correlation: What is the correlation between BINs and taxonomy? A good way to visualize this would be a histogram with the number of BINs on the x-axis and the number of species on the y-axis. This would help illustrate the relationship between BIN clustering and species-level taxonomy.

**Authors comments:**

We have added a graph as suggested, see graph below and Line 590-591 (citing S4 Fig).

**S4 Fig:** **BIN to species ratio regression analysis**. The relationship between the number of BINs assigned to records and the number of species morphologically identified and assigned to species name, with the black line indicating the 1:1 ratio (BIN to Species).

**Reviewer #1: Comment 5**

Accessibility of Barcode Data: What is the level of accessibility for barcode data across plants, animals, and fungi? Please include a figure showing the percentage of barcodes that are private versus public for each of these three major groups.

**Authors comments:**

We have updated the paper and generated the graph as suggested, see graph below and Line 359-361 and 872-874 (citing S1 Fig).

**S1 Fig: The comparison of public and private South African BOLD records.** Highlighting the total number of South African records on BOLD constituted by public and private records. * Other organisms with COI barcode data such as bacteria, protista.

**Reviewer #1: Comment 6**

Page 17 (now page 18), Table 1: Please clarify what is meant by "unique BINs." Does this refer to BINs that contain exclusively South African records?

**Authors comments:**

Page 18: The text in the table has been adjusted for clarity. We have removed the term unique and just referred to them as BINs.

**Reviewer #1: Comment 7**

Page 20, Lines 436-440 (now page 21, Line 447-452): Many barcodes are being submitted directly to GenBank without being deposited in BOLD first or in parallel. This is problematic because many of these sequences lack easily accessible metadata. Please discuss this issue.

**Authors comments:**

We have updated the text as suggested, see Line 447-461.

**Reviewer #1: Comment 8**

Page 37, Line 839 (now page 39, line 892-895): To enable real-time monitoring of how the reference library is being populated, you could use the checklist tool available in BOLD. If you upload the checklists you created (Tables S1-S4), BOLD can generate automatic progress reports and hit lists of species that still need to be barcoded. Currently, these lists and reports would be based mainly on South African barcode data, since barcoding efforts in other southern African countries remain limited. However, the new version of BOLD (BOLD5) may allow geographic filtering, enabling a more refined analysis focused solely on South African specimens. This is worth mentioning in the discussion. Having these checklists available would be highly beneficial for anyone interested in assessing barcode coverage for specific taxonomic groups.

**Authors comments:**

We have updated the text to include the BOLD tools as suggested, see Line 892-895.

**Reviewer #1: Comment 9**

Inclusion of GenBank Accession Numbers:

How many barcodes are being published without GenBank accession numbers? This is a widespread issue in the literature, even in high-impact journals, as many researchers are unaware that barcodes in BOLD can be easily deleted, whereas data in GenBank have a permanent status. Another concern is the use of publicly available but unpublished barcodes in BOLD, which lack GenBank accession numbers. In such cases, the taxonomic identifications may still be preliminary. Researchers should be encouraged to contact project managers before using unpublished barcodes to verify their accuracy. This issue is worth discussing in the manuscript.

**Authors comments:**

We have generated a graph that illustrates the number of barcode records that do not have GenBank numbers. Further we have addressed the issue in the text in terms of the use of unverified barcode data that is not yet published for species delimitation. See graph (S2 Fig) below and Line 451-452, 891-892.

**S2 Fig:** Proportion of barcode records containing GenBank accession numbers compared to those without, across taxonomic groups.

**Reviewer #2: The authors of this study attempted to assess current progress of DNA barcode coverage in South Africa. While the volume of data that the authors have processed in their analysis is significant, I found some of their methods to be either flawed or confusing, and their results were difficult to interpret.**

**Reviewer #2: Comment 1**

For starters, a major component of their study is a gap analysis that compared species represented in BOLD to those included on verified checklists. The problem with this approach is that while it works particularly well in groups where the number of described species is very comprehensive (e.g., in some vertebrates, like birds in particular), for taxonomically under-studied groups like insects, it's more like comparing two unknown variables. This uncertainty is captured in line 356 (now 366): "approximately 38% of South African species MAY HAVE already been barcoded". They MIGHT have, or the list of species might only represent a small fraction of the actual number of species. So while it's clear that there is a 'gap', it's not clear what the gap is (i.e., do taxonomists need to review specimens on BOLD and get more assigned to species, or do we need biologists out in the field collecting more samples?). Ultimately, using a list of species names to assess progress for records that are largely unidentified at the species level doesn't seem likely to work.

**Authors comments:**

Thank you very much for your comments. We agree that due to the lack of comprehensive checklists for some groups the analysis might not work particularly well for these specific comparisons. However, we hope that this paper will highlight both of these instances as they support the need for growing libraries and taxonomic capacity as there is 1) lack of capacity to verify specimens and 2) the unavailability of checklists to verify all species names. Using the currently known checklist, we are able to indicate what has been barcoded with regard to what we know to exist currently, even though we are aware that there are far more that exist than we have on the list for some taxon groups, particularly for insects (as seen in S11 Table) and marine inverts. We have therefore updated our text in the paper to indicate this and provide more clarity. See Line 365-370 and 541-547.

**Reviewer #2: Comment 2**

Ignoring the first major conceptual issue, the results reported in the first paragraph of the Results section and Table 1 are still very confusing. Summarizing this amount of information is not easy, but I had to re-read the first paragraph several times to understand what the authors were reporting, and some elements of the Table I never understood. For example, how is number of BINs per species a different measure from BIN to species ratio? I couldn't figure out where numbers were being derived from for the former.

**Authors comments:**

Thank you for the comment, we have updated the Table and deleted the column (page 18 column 4 of Table 1). It was calculated similarly to the ratio using incorrect values, also it was meant to serve a different purpose which was never clearly outlined in the paper therefore the whole idea ended being removed from the paper.

**Reviewer #2: Comment 3**

The fact that their analysis of BOLD focused on records rather than sequences also surprised me. Perhaps this was necessary because the authors were interested in more than just COI for example, but with the major focus on BINs, narrowing the scope to sequences might have been more straightforward. Either way, I think it should have been easy to eliminate records with no sequences at all from their analysis - I don't know what value was brought by including them.

**Authors comments:**

We appreciate this comments, this study was not solely focused on BIN assignments. Including records without sequences (metadata captured but sequences failed or still to be uploaded) adds value to the analysis by providing a comprehensive overview of all available specimen data (occurrence of species in South Africa), allowing the identification of gaps to direct future work. Despite lacking sequences, these records reveal which species have been collected and provide occurrence data that might not necessarily be in museum collections but would add records to GBIF. Additionally, these records represent specimens that might still be awaiting sequencing or those excluded due to failed quality standards, which highlights potential challenges within the DNA barcoding workflow requiring attention.

**Reviewer #2: Comment 4**

The comparison to GenBank confused me a bit too. From the methods, it reads as though all sequences were downloaded regardless of what gene or region they belonged to, is that correct? I didn't fully understand what records in BOLD were being compared to GenBank either. In the methods it sounds like the comparison is between all sequences from GenBank and a portion of those sequences that have been mined to BOLD? But then on page 20 (now 21), sweeping statements are made about the number of sequences held in the two platforms and how many more GenBank has. But this is all genes? Or just certain markers? Or just comparing to the ones that have been mined? It's not clear what point you're trying to get at, but even the numbers reported (e.g., in Figure 3) are hard to interpret. How can only 9,804 animal sequences be associated with BOLD based on the numbers reported elsewhere? As with some of the figures/tables, the caption is very simple and it not fully explaining the figure.

Authors comments:

Thank you for the comment. We have realised that the text was not clear and have updated this in the paper. The comparison was for GenBank records of IBOL-approved barcode genes including those records that meet the BOLD standard versus sequences mined by BOLD from GenBank. We have updated the figure to indicate the total number of records in both datasets whilst highlighting the subset of records that are shared by both databases. See Figure 3 below and figure caption in Line 447-461.

**Fig 3. The number of published South African DNA sequence records using the iBOL-approved barcode gene regions for animals (COI), fungi (ITS), and plants (matK, rbcLa): dark grey represents total records on BOLD, mid grey records mined from GenBank by BOLD (shared records) and light grey total records on GenBank.**

**Reviewer #2: Comment 5**

Given the different barcoding challenges still being faced broadly by other groups, such as plants and fungi, incorporating them into one analysis alongside animals is all the more challenging. Consequently, the authors end up devoting space in the Discussion commenting on general issues that go beyond the scope of progress in South Africa specifically.

**Authors comments:**

The paper is a broad study looking at all the organisms barcoded in South Africa. It is important that all South African information on BOLD be reported on. At times general statements/paragraphs were added to highlight a specific point, however efforts were made to drive the South African context from those points. The authors will amend the general statements/paragraphs where it’s appropriate and focus on specific information.

**Reviewer #2: Comment 6**

In addition to the issues described above, there were a few oversights and typographic errors throughout the paper. For example:

-Line 186 (now Line 190): "rbcLa" is spelled incorrectly

**Authors comments:**

“rcbLa” was corrected to “rbcLa”, see Line 190.

-Line 235 (now Line 240): "DNA" is missing from "DNA Data Bank of Japan"

**Authors comments:**

“DNA” was added as per suggestion in Line 240.

-Lines 275-276 (now Line 283): BOLD does not include a general keyword search. Phrases are searched for inside of specific fields. I assume it is meant that this term was searched in the 'Geography' field, but please specify.

**Authors comments:**

The sentence was amended as per suggestion in Line 283.

-Lines 328-332 (now Line 337-341): The publications were not separated into five distinct categories, rather they were categorized according to two distinct sets of criteria: first, those that generated barcodes or not, and secondly, by environment. Either way, the second categories used by the authors is not really a separate category.

**Authors comments:**

Categories were changed into subsections in Line 337-341.

-Line 349 (now Line 384): Unless I've missed it, I don't think the authors indicate the date that records were downloaded from BOLD in the body of the manuscript, which is important since the database is constantly growing. I think it only shows up in the caption for Table 1.

**Authors comments:**

The date benchmarking the study is mentioned four times: Line 246 (under aims), Line 385 (under Table 1 caption), Line 514 (under second paragraph of discussion) and Line 523 (under third paragraph of discussion).

-Line 508-509 (now Line 523-533): I think this might be phrased incorrectly. Anything without a family-level classification is also going to be missing a species name, no? So shouldn't these proportions be accumulative?

**Authors comments:**

It is correct that anything without a family-level classification is going to be missing a genus and species name. In this case, 26% of records had differing levels of classification categories from Kingdom up to order. The rest of the data was accounted for by adding 74% and actual numbers were included for better comprehension. See Line 523-533.
