# Supplementary material for: An overview of DNA barcoding of biodiversity in South Africa
Source: PLoS One. 2026 Apr 21;21(4):e0345173. doi: 10.1371/journal.pone.0345173 (PMC13098986; doi:10.1371/journal.pone.0345173)
Supplement: Attachment — Submitted filename: 20250111_PLODONE_review.docx [file pone.0345173.s016.docx]

**Reviewer Summary – PLOS ONE**

**"An overview of DNA barcoding of biodiversity in South Africa"**

**Overall assessment**
This manuscript provides a comprehensive and timely synthesis of DNA barcoding efforts in South Africa by integrating a literature review with a large-scale analysis of public sequence repositories (BOLD and GenBank). The topic is relevant, the dataset is extensive, and the study fits well within the scope of PLOS ONE. The work is technically sound; however, several methodological choices and interpretations require clarification to improve transparency, reproducibility, and robustness of the conclusions.

**Key issues requiring attention**

1. **Clarity of study design and terminology**
   The manuscript should more clearly define whether it represents a review, a data-driven gap analysis, or a hybrid synthesis, particularly in the abstract and introduction. Several terms (e.g. “species barcode records,” “taxonomic representation”) are potentially ambiguous and should be defined precisely to avoid misinterpretation.
2. **Methodological transparency and reproducibility**
   Greater detail is needed regarding data retrieval strategies from BOLD and GenBank (search fields, filters, handling of incomplete metadata), quality control procedures, and taxonomic standardisation (including synonym handling and reference taxonomies). Differences in checklist sources and compilation methods among plants, fungi, and animals should be acknowledged as a potential source of bias in cross-taxon comparisons.
3. **Treatment of taxonomic uncertainty**
   The decision to treat records with qualifiers such as *cf.*, *aff.*, or *nov.* as valid species names, and to collapse infraspecific taxa to species level, is unconventional and may inflate estimates of barcode coverage. These choices require clearer justification and discussion of their implications for interpreting taxonomic gaps.
4. **Geographic analyses and institutional contributions**
   Assigning provincial centroids to records lacking GPS coordinates and restricting analyses to BIN-assigned records may bias geographic patterns, particularly for underrepresented taxa such as plants and fungi. The categorisation of institutional contributions (local, regional, international) based on storage location also requires clarification, given possible mismatches with sampling locality and data ownership.
5. **Interpretation of BIN-based results**
   Estimates of barcoding completeness based on the assumption that each BIN represents a distinct species should be presented more cautiously, as this relationship is taxon-dependent. BIN splits and merges should not be interpreted solely as taxonomic deficiencies, as they may also reflect biological processes or methodological thresholds inherent to BIN assignment.

**Conclusion**
Addressing these points—primarily through clearer methodological descriptions, cautious interpretation of BIN-based metrics, and explicit discussion of limitations—would substantially strengthen the manuscript and its contribution to understanding DNA barcoding progress and gaps in South Africa.
