# Supplementary material for: An overview of DNA barcoding of biodiversity in South Africa
Source: PLoS One. 2026 Apr 21;21(4):e0345173. doi: 10.1371/journal.pone.0345173 (PMC13098986; doi:10.1371/journal.pone.0345173)
Supplement: Attachment — Submitted filename: Response_to_Reviewers_auresp_2.docx [file pone.0345173.s018.docx]

**Response**

**Abstract**

- **Line 133-** “BINS” should be corrected to “BINs.”
  - ***Updated:*** *“S” changed to smaller case “s”.* ***See line 133***
- **Line 137-** The standard usage of “cytochrome oxidase subunit I (COI)” is “cytochrome c oxidase subunit I (COI).”
  - ***Updated:*** *“cytochrome oxidase subunit I (COI)” changed to “cytochrome c oxidase subunit I (COI).”* ***See*** ***line 137***
- **Lines 138*-*** The phrase “Discrepancies between GenBank records and the records mined by BOLD from GenBank…” repeats “records” and “GenBank,” which reduces clarity. Consider rephrasing to make it more concise and readable.
  - ***Updated:*** *phrase changed to* *“Discrepancies between GenBank records and those mined by BOLD indicated that…”* ***See*** ***line 138***
- **Lines 142-** The phrase “resolve BIN assignment for inconsistent taxonomic assignments” is unclear. Rewriting it as “resolve inconsistencies in BIN assignments, particularly for underrepresented groups” would improve clarity and precision.
  - ***Updated:*** *rewritten “resolve BIN assignment for inconsistent taxonomic assignments” to “resolve inconsistencies in BIN assignments, particularly for underrepresented groups”* ***See*** ***line 142***

**Introduction**

- The section is overly long and at times diffuse, particularly in lines 167-213 where extensive background on taxonomy history, the Global Taxonomy Initiative, species concepts, and ICZN recommendations may exceed what is necessary to frame a review of DNA barcoding progress in South Africa.
  - ***Updated:*** *lines 167-213 have been rephrased and streamlined make them more concise and shorter.* ***See line*** ***167-192***
- For citing in the text follow the journal guideline, such as Hebert et al. (2003) not Hebert et al. [12] line 505.
  - ***Updated:*** *all the citations in text were changed from the format “Hebert et al. [12] to “Hebert et al. (2003) [12]. For all such citations throughout the manuscript.”*

**DNA barcoding for monitoring biodiversity**

Several points would benefit from clarification or refinement-

- **Line 156-** The reference to “three of the 35 globally recognized biodiversity hotspots” lacks specificity. Please name the three hotspots explicitly to improve clarity and regional relevance.
  - ***Updated:*** *added the Cape Floristic Region, the Succulent Karoo, and the Maputaland-Pondoland-Albany corridor.* ***See line 155-156***
- **Line 157-** Percentages and absolute species numbers are used interchangeably, which may reduce interpretability. Consider using one consistently, or explicitly justify the use of both.
  - ***Updated:*** *the text was updated for clarity and to reduce the inconsistency, we aim to use both as appropriate.* ***See line 158-159***
- **Line 170-** less than 14% of global species diversity has been documented is dependent on uncertain estimates. This claim should be qualified using cautious language (e.g. “estimated” or “likely”).
  - ***Updated:*** *the text for simplicity.* ***See 167-168***
- **Line 187-** While standard barcode regions are listed, their known limitations are not mentioned. Briefly noting constraints-such as lower discriminatory power in plants and fungi-would provide a more balanced overview.
  - ***Updated:*** *added text “However, there is lower discriminatory power in some taxon groups such as plants and fungi as some taxa require additional secondary markers for more accurate barcode species delimitation.”* ***See line 184-186***
- **Line 193**- The controversy surrounding DNA barcoding is acknowledged but not explained. Adding a brief clause summarising key criticisms (e.g. reliance on single-locus data) would strengthen the discussion.
  - ***Updated:*** *the text and added reference. Meyer, C. P., & Paulay, G. (2005). DNA Barcoding: Error Rates Based on Comprehensive Sampling. PLOS Biology, 3(12), e422.* [*https://doi.org/10.1371/JOURNAL.PBIO.0030422*](https://doi.org/10.1371/JOURNAL.PBIO.0030422)*.* ***See line 186-189***
- **Line 206-** The sentence is overly long and grammatically awkward. Splitting it into two sentences would improve clarity.
  - ***Updated:*** *sentence is split into two.* ***See line 195-199***
- **Line 216-** “Continents” should be revise to “continents.”
  - ***Updated:*** *corrected.* ***See line 216***
- **Line 248-** Objectives (1) and (2) partially overlap. Refining them to ensure each addresses a distinct analytical component would improve conceptual clarity.
  - ***Updated:*** *the objectives have been adjusted for specificity.* ***See line 236-239***

**Materials and Methods**

- **Line 259-** The SANBI national plant checklist is cited, but the date of data download is not provided. Please add the exact download date, as is done for other datasets.
- ***Updated:*** *date added.* ***See line 249-250***
- ***Line 262-*** Multiple fungal data sources are listed, but the strategy for integrating them is unclear. Clarify whether sources were merged, prioritised, or cross-validated, and explain how conflicting names or classifications were resolved.
- ***Updated****: added the following paragraph – “After data acquisition, each list was sorted alphabetically and species names were cross-compared to identify and remove exact duplicates. Author citations were then removed, and species names were concatenated into a single standardised string comprising the genus and specific epithet, including subspecies, varieties, and unique numerical identifiers, to enable further duplicate detection. A fuzzy matching approach was subsequently applied to identify potential typographical and spelling inconsistencies, which were verified manually. In cases of conflict, nomenclature from authoritative sources, including Index Fungorum and published checklists, was adopted. This process resulted in the final fungal checklist (S2 Table) used in the study.”* ***See line 257-265***
- **Line 264-** The phrase “Index Fungorum () database” contains empty parentheses. These should be removed or completed.
- ***Updated****: deleted ().* ***See line 253***
- **Line 266-** Using a single search term (“fungi South Africa”) is overly simplistic and likely incomplete, risking omission of taxa due to synonymy, spelling variation, or missing locality metadata.
- ***Updated:*** *we have updated the search as it was two terms “fungi” and “South Africa” and noted the limitation of the methodological approach that it could have missed data that did not list the country as source but rather specific localities and did not have any taxonomic classification.* ***See line 255-257***
- **Line 268-** Animal checklists are attributed to “taxon experts,” but criteria for expert selection and validation are not specified. Indicate whether these lists were peer-reviewed or cross-checked against recognised taxonomic authorities.
- ***Updated:*** *the following text was added – “These checklists are compiled under the guidance of a national checklist committee and are verified and cross-referenced before publication.”* ***See line 266-267***
- **Line 273-** Treating records containing qualifiers such as “cf.”, “aff.”, or “nov.” as valid species names is problematic and may inflate species counts, biasing gap analyses.
- ***Updated:*** *we have updated the text. While these may conflate the species numbers and add bias, we used checklist verified by experts working on these groups.* ***See line 273-274***
- **Lines 276-278-** The statement that “problematic records were individually checked” lacks quantification. Please indicate how many records required manual correction.
- ***Updated:*** *Number of records has been included.* ***See line 277***
- **Line 282-** Restricting searches to records labelled “South Africa” in BOLD may miss entries with incomplete or erroneous geographic metadata. This limitation should be acknowledged.
- ***Updated****: to note the limitation.* ***See line 283-284***
- **Line 294-** The treatment of uncertain species identifications as valid species is reiterated. To avoid redundancy, refer back to the justification provided earlier (line 273).
- ***Updated:*** *repetition was removed and sentence amended.* ***See line 295-296***
- **Line 301-** Key terms such as “distinct records” and “iBOL-compliant” are not operationally defined and should be clearly specified.
- ***Updated:*** *the terms were defined as follows – “Number of distinct records (unique voucher specimens)” and “iBOL-compliant (DNA sequences that meet the standards, policies, and protocols established for DNA barcodes as outlined by iBOL).”* ***See line 300-302***
- **Line 303-** Barcode Index Numbers (BINs) are COI-specific. Counting BINs across barcode regions may therefore be conceptually misleading. Clarify explicitly that BIN-based analyses apply only to animal COI records.
- ***Updated:*** *the text has been clarified.* ***See line 304***
- **Line 306-** BIN splits and merges are interpreted as indicators of taxonomic issues without consideration of misidentification. Please clarify whether records were screened for misidentifications.
- ***Updated:*** *we have updated the text to include misidentifications as another option. We did not verify identifications of split and merges in our analyses of the large dataset.* ***See line 310***
- **Line 316-** Analyses are restricted to records assigned BINs, excluding potentially informative non-BIN barcode data. Justify this exclusion and discuss its implications, particularly for plant and fungal datasets.
- ***Updated:*** *we did not include non-BIN COI records in the analysis as we did not do phylogenetic analysis for other gene regions to verify their species identification. We agree that this may result in an underestimation and will indicate this in the methodology.* ***See line 318-320***
- **Line 328-** The study is not described as a systematic or scoping review. Please justify the exclusion of publications prior to 2004 and clarify whether earlier relevant studies exist.
- *Updated: Sentence adjusted for justification. The analysis only included records after the 2003 publication on DNA barcoding. While there could be other data that used these gene regions prior to 2003, we only include papers that used the term barcoding, text has been updated.* ***See line 332***
- **Line 333-** The term “[molecular term]” appears in a third brackets without explanation. Please clarify its purpose or remove it.
- ***Updated:*** *we deleted the word term from the brackets.* ***See line 336***
- **Line 349**- The comparison between GenBank and BOLD records lacks methodological detail. Specify how overlap was assessed (e.g. accession numbers, species names, markers) and indicate whether discrepancies were quantified or only qualitatively described.
- ***Updated:*** *methodology was updated with the following sentence – “The total number of BOLD records annotated with the tag “Mined from GenBank, NCBI” in the “institution_storing” field was compared to the total number of records retrieved directly from GenBank, filtered by standard barcode markers (COI for animals, matK and rbcLa for plants, and ITS for fungi).”* ***See line 353-356***

**Results**

- **Lines 356-358-** The presentation of species counts and percentages is clear, but consistency would improve readability. Consider using either absolute numbers or percentages uniformly, or presenting both in a parallel structure.
- ***Updated:*** *the counts and percentages were amended for consistency.* ***See line 362-363***
- **Lines 359-361-** The distinction between public and private records is clearly described. However, the estimate of approximately 13 million global records would benefit from a citation or a brief note indicating when this figure was retrieved, as database content changes over time.
- ***Updated:*** *the date of when the figures were retrieved was added.* ***See line 365***
- **Lines 362-366-** The rationale for analysing only public records is appropriate but could be stated more succinctly.
- ***Updated:*** *the sentence was updated to be more succinct.* ***See line 368-369***
- **Lines 372-374-** The explanation for the absence of BIN assignments for certain unique names is reasonable.
- ***Updated:*** *the sentence was updated as follows – “the 3,832 unique names not assigned to BINs likely reflect non-compliance with iBOL barcode standards or missing sequence data for the corresponding specimens.”* ***See line 378-379***
- **Lines 382-384-** Fungi and plants are identified as underrepresented groups. For plants, the text notes that the two-gene barcode system (matK and rbcLa) could not be fully captured. It would strengthen the Results section to briefly explain why this limitation exists (e.g., BOLD platform constraints, incomplete marker coverage, or data formatting issues).
- ***Updated:*** *the following sentence was added – “It is important to note that the Barcode Index Number (BIN) system was developed specifically for clustering animal COI sequences and is not compatible with plant barcodes (matK and rbcLa) or fungal ITS regions.”* ***See line 390-393***

Table 1- The absence of BINs for fungi is a notable result and should be more clearly explained in the Results text. Explicitly state that BINs are based on COI sequences and are therefore not applicable to fungal ITS barcodes (this could also be reinforced with a table footnote).

- ***Updated:*** *the following footnote was added – “BINs are based solely on COI sequences and are threfore not applicable to fungal ITS and plants matK and rbcLa.”* ***See line 400***

Similarly, the presence of only 119 BINs for 3,469 plant species requires clarification. Briefly explain in the Results that BINs are largely incompatible with standard plant barcoding markers, which accounts for the low BIN representation despite relatively high species counts.

- ***Updated:*** *the following sentence was added – “Furthermore, despite plants having relatively high species count, the low 119 BINs highlight the incompatibility of the BIN system to standard plant barcoding markers.* ***See line 388-389***

**Discussion**

- Paragraphs are very long (e.g., lines 502-554, 734-762), often covering multiple points. Suggest breaking long paragraphs into smaller, thematic paragraphs (e.g., separate methods/technical challenges, applications, capacity-building, and funding).
- ***Updated:*** *the paragraphs were separated into smaller ones according to thematic category.* ***See line 515-519, 742-743, 747, 761***
- Data presentation is sometimes confusing, mixing percentages and absolute numbers. Example- lines 518-520- “animal records have increased by 890%, insects 973%”-readers may benefit from absolute numbers for context.
- ***Updated:*** *absolute numbers were added.* ***See line 521-523***
- **Line 553-** OUT should be OTU
- ***Updated:*** *OUT changed to OTU.* ***See line 555***
- **Line 625-** COI is used for plants; previously, it was clarified that matK and rbcLa are standard. Consider briefly clarifying the historical context versus current standards.
- ***Updated:*** *the sentence was clarified as follows – “In contrast, plants were included because BINs have been assigned, largely derived from historical experimental sequencing projects, mitochondrial genome studies, or GenBank-imported data using the COI gene, which is not the standard barcode marker for plants.”* ***See line 633-635***

**Lines 858-860-** Discussion of plant BINs is a bit unclear; needs stronger linkage between issue (COI misassignment) and recommended solution.

- ***Updated:*** *the sentence was amended as follows – “However, for BOLD to assign BINs to a sequence, it must meet set requirements, including representation by a single locus, a requirement that the experimental COI gene in plants did not meet.”* ***See line 859-861***
- The discussion refers to multiple figures and tables (S3, S5, S8, S11, Fig 2, Fig 3, Fig 6), but these could be better integrated into the narrative with key takeaways highlighted.
- ***Updated:*** *the following figures and tables were integrated into the narrative and removed all together from being part of the manuscript: S3 Fig, S5 Fig, S3 Table, S5 Table, S7 Table, S9 Table, S10 Table and S11 Table. S5 Table will be provided with the manuscript as part of the main dataset. S8 Table was kept for visual effects of the results which were extensively discussed.* ***See last page (49)***
- Consider summarizing major figure points in the text rather than just referencing them.
- ***Updated:*** *the following major figure points were integrated into the narratives and removed from the manuscript: Fig 2, Fig 3, Fig 6). Fig 2 was converted into S1 Fig due to its visual effects of showing all the sequence records associated with South Africa.* ***See page 24, 33 and last page (49)***
- Some sentences are dense and could be simplified without losing meaning. Examples- lines 805-811, lines 833-841. Shorter sentences would improve readability.
- ***Updated:*** *the sentences have been amended.* ***See line 841-846***
- Clarify the COI limitation discussion- why COI is used, where it fails, and alternatives.
- ***Updated:*** *the following paragraph was added – “The COI gene region is widely used for animal DNA barcoding because it is relatively conserved within species but variable between species [18]. However, its effectiveness is not universal across all taxa; for example, the Folmer region often performs poorly in amphibians [107]. In such cases, alternative markers, including Cytb, 12S, and 16S, are used to complement or replace COI [82]. In plants, matK and rbcLa are commonly employed, though rbcLa amplifies easily but poorly discriminates species, while matK discriminates well but is difficult to amplify, especially in non-angiosperms; alternative chloroplast spacers such as trnH-psbA, atpF-atpH, and psbK-psbI are sometimes used [108]. For fungi, ITS is the primary barcode, with TEF-α, RPB, and β-tubulin serving as alternative markers when necessary [109].”* ***See line 867-876***
- Ensure all acronyms (e.g., HTS, eDNA, SABOL, FBIP) are consistently defined at first use.
- ***Updated:*** *the acronyms have been defined at first use. The only one not defined at first use was HTS.* ***See line 413-414***

**Conclusion**

- The conclusion would be stronger if it briefly reiterated one or two headline results (e.g., overall barcoding coverage, major taxonomic or geographic gaps) to anchor the recommendations in the study’s core findings.

Consider reducing repetition of “should” by varying sentence structure.

- ***Updated:*** *the conclusion has been amended.* ***See line 901-904***
