# Supplementary material for: An overview of DNA barcoding of biodiversity in South Africa
Source: PLoS One. 2026 Apr 21;21(4):e0345173. doi: 10.1371/journal.pone.0345173 (PMC13098986; doi:10.1371/journal.pone.0345173)
Supplement: Peer review history

# Peer review: Decision Letter 0

**DOI:** 10.1371/journal.pone.0345173.r001

**Authors:** Mehrdad Hajibabaei

4 Apr 2025

Dear Dr. Kgatla,

Thank you for submitting your manuscript to PLOS ONE. After careful consideration, we feel that it has merit but does not fully meet PLOS ONE’s publication criteria as it currently stands. Therefore, we invite you to submit a revised version of the manuscript that addresses the points raised during the review process.

==============================

I have obtained two reports from reviewers who are very familiar with different aspects of this study. Based on these reports and my own assessment, I see potential for publication. However, the manuscript requires substantial revisions, some of which require reanalysis, additional analysis, rewriting, etc. as stated by the reviewers. If you decide to revise the manuscript, please note that I will need to seek another round of review to ensure the revised manuscript addresses the concerns raised.

==============================

Please submit your revised manuscript by May 19 2025 11:59PM. If you will need more time than this to complete your revisions, please reply to this message or contact the journal office at plosone@plos.org. When you're ready to submit your revision, log on to https://www.editorialmanager.com/pone/ and select the 'Submissions Needing Revision' folder to locate your manuscript file.. When you're ready to submit your revision, log on to https://www.editorialmanager.com/pone/ and select the 'Submissions Needing Revision' folder to locate your manuscript file.. When you're ready to submit your revision, log on to https://www.editorialmanager.com/pone/ and select the 'Submissions Needing Revision' folder to locate your manuscript file.. When you're ready to submit your revision, log on to https://www.editorialmanager.com/pone/ and select the 'Submissions Needing Revision' folder to locate your manuscript file.

- A rebuttal letter that responds to each point raised by the academic editor and reviewer(s). You should upload this letter as a separate file labeled 'Response to Reviewers'.
- A marked-up copy of your manuscript that highlights changes made to the original version. You should upload this as a separate file labeled 'Revised Manuscript with Track Changes'.
- An unmarked version of your revised paper without tracked changes. You should upload this as a separate file labeled 'Manuscript'.

If you would like to make changes to your financial disclosure, please include your updated statement in your cover letter. Guidelines for resubmitting your figure files are available below the reviewer comments at the end of this letter.

If applicable, we recommend that you deposit your laboratory protocols in protocols.io to enhance the reproducibility of your results. Protocols.io assigns your protocol its own identifier (DOI) so that it can be cited independently in the future. For instructions see: https://journals.plos.org/plosone/s/submission-guidelines#loc-laboratory-protocols. Additionally, PLOS ONE offers an option for publishing peer-reviewed Lab Protocol articles, which describe protocols hosted on protocols.io. Read more information on sharing protocols at . Additionally, PLOS ONE offers an option for publishing peer-reviewed Lab Protocol articles, which describe protocols hosted on protocols.io. Read more information on sharing protocols at . Additionally, PLOS ONE offers an option for publishing peer-reviewed Lab Protocol articles, which describe protocols hosted on protocols.io. Read more information on sharing protocols at . Additionally, PLOS ONE offers an option for publishing peer-reviewed Lab Protocol articles, which describe protocols hosted on protocols.io. Read more information on sharing protocols at https://plos.org/protocols?utm_medium=editorial-email&utm_source=authorletters&utm_campaign=protocols....

We look forward to receiving your revised manuscript.

Kind regards,

Mehrdad Hajibabaei

Academic Editor

PLOS ONE

Journal requirements:

When submitting your revision, we need you to address these additional requirements.

1.  Please ensure that your manuscript meets PLOS ONE's style requirements, including those for file naming. The PLOS ONE style templates can be found at

https://journals.plos.org/plosone/s/file?id=wjVg/PLOSOne_formatting_sample_main_body.pdf  and  and  and  and

https://journals.plos.org/plosone/s/file?id=ba62/PLOSOne_formatting_sample_title_authors_affiliations.pdf

2. When completing the data availability statement of the submission form, you indicated that you will make your data available on acceptance. We strongly recommend all authors decide on a data sharing plan before acceptance, as the process can be lengthy and hold up publication timelines. Please note that, though access restrictions are acceptable now, your entire data will need to be made freely accessible if your manuscript is accepted for publication. This policy applies to all data except where public deposition would breach compliance with the protocol approved by your research ethics board. If you are unable to adhere to our open data policy, please kindly revise your statement to explain your reasoning and we will seek the editor's input on an exemption. Please be assured that, once you have provided your new statement, the assessment of your exemption will not hold up the peer review process.

3. We note that Figure 1 in your submission contain [map/satellite] images which may be copyrighted. All PLOS content is published under the Creative Commons Attribution License (CC BY 4.0), which means that the manuscript, images, and Supporting Information files will be freely available online, and any third party is permitted to access, download, copy, distribute, and use these materials in any way, even commercially, with proper attribution. For these reasons, we cannot publish previously copyrighted maps or satellite images created using proprietary data, such as Google software (Google Maps, Street View, and Earth). For more information, see our copyright guidelines: http://journals.plos.org/plosone/s/licenses-and-copyright.

We require you to either (1) present written permission from the copyright holder to publish these figures specifically under the CC BY 4.0 license, or (2) remove the figures from your submission:

a. You may seek permission from the original copyright holder of Figure 1 to publish the content specifically under the CC BY 4.0 license.

We recommend that you contact the original copyright holder with the Content Permission Form (http://journals.plos.org/plosone/s/file?id=7c09/content-permission-form.pdf) and the following text:

“I request permission for the open-access journal PLOS ONE to publish XXX under the Creative Commons Attribution License (CCAL) CC BY 4.0 (http://creativecommons.org/licenses/by/4.0/). Please be aware that this license allows unrestricted use and distribution, even commercially, by third parties. Please reply and provide explicit written permission to publish XXX under a CC BY license and complete the attached form.”

Please upload the completed Content Permission Form or other proof of granted permissions as an ""Other"" file with your submission.

In the figure caption of the copyrighted figure, please include the following text: “Reprinted from [ref] under a CC BY license, with permission from [name of publisher], original copyright [original copyright year].”

b. If you are unable to obtain permission from the original copyright holder to publish these figures under the CC BY 4.0 license or if the copyright holder’s requirements are incompatible with the CC BY 4.0 license, please either i) remove the figure or ii) supply a replacement figure that complies with the CC BY 4.0 license. Please check copyright information on all replacement figures and update the figure caption with source information. If applicable, please specify in the figure caption text when a figure is similar but not identical to the original image and is therefore for illustrative purposes only.

The following resources for replacing copyrighted map figures may be helpful:

USGS National Map Viewer (public domain): http://viewer.nationalmap.gov/viewer/

The Gateway to Astronaut Photography of Earth (public domain): http://eol.jsc.nasa.gov/sseop/clickmap/

Maps at the CIA (public domain): https://www.cia.gov/library/publications/the-world-factbook/index.html and https://www.cia.gov/library/publications/cia-maps-publications/index.html

NASA Earth Observatory (public domain): http://earthobservatory.nasa.gov/

Landsat: http://landsat.visibleearth.nasa.gov/

USGS EROS (Earth Resources Observatory and Science (EROS) Center) (public domain): http://eros.usgs.gov/#

Natural Earth (public domain): http://www.naturalearthdata.com/

4. Please upload a copy of Supporting Information Files which you refer to in your text on pages 44 and 45.

[Note: HTML markup is below. Please do not edit.]

Reviewers' comments:

Reviewer's Responses to Questions

**Comments to the Author**

1. Is the manuscript technically sound, and do the data support the conclusions?

Reviewer #1: Yes

Reviewer #2: No

**********

2. Has the statistical analysis been performed appropriately and rigorously?

Reviewer #1: Yes

Reviewer #2: No

**********

3. Have the authors made all data underlying the findings in their manuscript fully available?

The PLOS Data policy requires authors to make all data underlying the findings described in their manuscript fully available without restriction, with rare exception (please refer to the Data Availability Statement in the manuscript PDF file). The data should be provided as part of the manuscript or its supporting information, or deposited to a public repository. For example, in addition to summary statistics, the data points behind means, medians and variance measures should be available. If there are restrictions on publicly sharing data—e.g. participant privacy or use of data from a third party—those must be specified.requires authors to make all data underlying the findings described in their manuscript fully available without restriction, with rare exception (please refer to the Data Availability Statement in the manuscript PDF file). The data should be provided as part of the manuscript or its supporting information, or deposited to a public repository. For example, in addition to summary statistics, the data points behind means, medians and variance measures should be available. If there are restrictions on publicly sharing data—e.g. participant privacy or use of data from a third party—those must be specified.

Reviewer #1: Yes

Reviewer #2: Yes

**********

4. Is the manuscript presented in an intelligible fashion and written in standard English?

Reviewer #1: Yes

Reviewer #2: No

**********

Reviewer #1: This paper presents a gap analysis of the level of coverage of DNA barcoding for South African biodiversity by comparing taxonomic checklists with DNA barcode records in BOLD and GenBank. Below are some comments for the authors to consider to improve their manuscript:

1. Page 24, Line 508: Could it be that most of the records lacking family, genus, or species names originate from insects collected using Malaise traps? Malaise trap samples often contain many dark taxa—micro-insects that are challenging to identify morphologically (e.g., micromoths). Using BOLD for their identification is difficult due to the relatively incomplete reference library for African fauna. Please discuss this point.

2. Visualizing Taxonomic Coverage: To better illustrate the level of coverage across taxonomic ranks, consider plotting a histogram. The x-axis could represent taxonomic ranks (order, family, genus, species), while the y-axis could show “representativity” (i.e., the percentage of taxa that have been barcoded at each rank). Additionally, for each rank, you could plot two bars: one representing the percentage of species barcoded and a second representing the percentage of species with more than five barcodes, which would provide insight into intraspecific sampling effort.

3. Insect Barcoding Coverage: Since most BOLD records correspond to insects—especially Diptera due to global Malaise trap campaigns—it would be useful to zoom in on South African insect data to assess potential disparities in barcode coverage among different insect orders.

4. BIN-Taxonomy Correlation: What is the correlation between BINs and taxonomy? A good way to visualize this would be a histogram with the number of BINs on the x-axis and the number of species on the y-axis. This would help illustrate the relationship between BIN clustering and species-level taxonomy.

5. Accessibility of Barcode Data: What is the level of accessibility for barcode data across plants, animals, and fungi? Please include a figure showing the percentage of barcodes that are private versus public for each of these three major groups.

6. Page 17, Table 1: Please clarify what is meant by "unique BINs." Does this refer to BINs that contain exclusively South African records?

7. Page 20, Lines 436-440: Many barcodes are being submitted directly to GenBank without being deposited in BOLD first or in parallel. This is problematic because many of these sequences lack easily accessible metadata. Please discuss this issue.

8. Page 37, Line 839: To enable real-time monitoring of how the reference library is being populated, you could use the checklist tool available in BOLD. If you upload the checklists you created (Tables S1-S4), BOLD can generate automatic progress reports and hit lists of species that still need to be barcoded. Currently, these lists and reports would be based mainly on South African barcode data, since barcoding efforts in other southern African countries remain limited. However, the new version of BOLD (BOLD5) may allow geographic filtering, enabling a more refined analysis focused solely on South African specimens. This is worth mentioning in the discussion. Having these checklists available would be highly beneficial for anyone interested in assessing barcode coverage for specific taxonomic groups.

9. Inclusion of GenBank Accession Numbers:

How many barcodes are being published without GenBank accession numbers? This is a widespread issue in the literature, even in high-impact journals, as many researchers are unaware that barcodes in BOLD can be easily deleted, whereas data in GenBank have a permanent status. Another concern is the use of publicly available but unpublished barcodes in BOLD, which lack GenBank accession numbers. In such cases, the taxonomic identifications may still be preliminary. Researchers should be encouraged to contact project managers before using unpublished barcodes to verify their accuracy. This issue is worth discussing in the manuscript

Reviewer #2: The authors of this study attempted to assess current progress of DNA barcode coverage in South Africa. While the volume of data that the authors have processed in their analysis is significant, I found some of their methods to be either flawed or confusing, and their results were difficult to interpret.

For starters, a major component of their study is a gap analysis that compared species represented in BOLD to those included on verified checklists. The problem with this approach is that while it works particularly well in groups where the number of described species is very comprehensive (e.g., in some vertebrates, like birds in particular), for taxonomically under-studied groups like insects, it's more like comparing two unknown variables. This uncertainty is captured in line 356: "approximately 38% of South African species MAY HAVE already been barcoded". They MIGHT have, or the list of species might only represent a small fraction of the actual number of species. So while it's clear that there is a 'gap', it's not clear what the gap is (i.e., do taxonomists need to review specimens on BOLD and get more assigned to species, or do we need biologists out in the field collecting more samples?). Ultimately, using a list of species names to assess progress for records that are largely unidentified at the species level doesn't seem likely to work.

Ignoring the first major conceptual issue, the results reported in the first paragraph of the Results section and Table 1 are still very confusing. Summarizing this amount of information is not easy, but I had to re-read the first paragraph several times to understand what the authors were reporting, and some elements of the Table I never understood. For example, how is number of BINs per species a different measure from BIN to species ratio? I couldn't figure out where numbers were being derived from for the former.

The fact that their analysis of BOLD focused on records rather than sequences also surprised me. Perhaps this was necessary because the authors were interested in more than just COI for example, but with the major focus on BINs, narrowing the scope to sequences might have been more straightforward. Either way, I think it should have been easy to eliminate records with no sequences at all from their analysis - I don't know what value was brought by including them.

The comparison to GenBank confused me a bit too. From the methods, it reads as though all sequences were downloaded regardless of what gene or region they belonged to, is that correct? I didn't fully understand what records in BOLD were being compared to GenBank either. In the methods it sounds like the comparison is between all sequences from GenBank and a portion of those sequences that have been mined to BOLD? But then on page 20, sweeping statements are made about the number of sequences held in the two platforms and how many more GenBank has. But this is all genes? Or just certain markers? Or just comparing to the ones that have been mined? It's not clear what point you're trying to get at, but even the numbers reported (e.g., in Figure 3) are hard to interpret. How can only 9,804 animal sequences be associated with BOLD based on the numbers reported elsewhere? As with some of the figures/tables, the caption is very simple and it not fully explaining the figure.

Given the different barcoding challenges still being faced broadly by other groups, such as plants and fungi, incorporating them into one analysis alongside animals is all the more challenging. Consequently, the authors end up devoting space in the Discussion commenting on general issues that go beyond the scope of progress in South Africa specifically.

In addition to the issues described above, there were a few oversights and typographic errors throughout the paper. For example:

-Line 186: "rbcLa" is spelled incorrectly

-Line 235: "DNA" is missing from "DNA Data Bank of Japan"

-Lines 275-276: BOLD does not include a general keyword search. Phrases are searched for inside of specific fields. I assume it is meant that this term was searched in the 'Geography' field, but please specify.

-Lines 328-332: The publications were not separated into five distinct categories, rather they were categorized according to two distinct sets of criteria: first, those that generated barcodes or not, and secondly, by environment. Either way, the second categories used by the authors is not really a separate category.

-Line 349: Unless I've missed it, I don't think the authors indicate the date that records were downloaded from BOLD in the body of the manuscript, which is important since the database is constantly growing. I think it only shows up in the caption for Table 1.

-Line 504-505: I think this might be phrased incorrectly. Anything without a family-level classification is also going to be missing a species name, no? So shouldn't these proportions be accumulative?

I appreciate that the authors are trying to make an important assessment of the local state of barcoding progress and think that this is a valuable exercise, but in its current form I found the paper was very challenging to digest. I think the authors need to carefully re-think their analysis and the presentation of the data, then narrow the scope of their discussion to better highlight key issues that they would like to see addressed in the future. If successful, it could perhaps become a model for others to follow.

**********

what does this mean?). If published, this will include your full peer review and any attached files.). If published, this will include your full peer review and any attached files.). If published, this will include your full peer review and any attached files.). If published, this will include your full peer review and any attached files.

If you choose “no”, your identity will remain anonymous but your review may still be made public.

**Do you want your identity to be public for this peer review?** For information about this choice, including consent withdrawal, please see our For information about this choice, including consent withdrawal, please see our For information about this choice, including consent withdrawal, please see our For information about this choice, including consent withdrawal, please see our Privacy Policy..

Reviewer #1: No

Reviewer #2: No

**********

[NOTE: If reviewer comments were submitted as an attachment file, they will be attached to this email and accessible via the submission site. Please log into your account, locate the manuscript record, and check for the action link "View Attachments". If this link does not appear, there are no attachment files.]

While revising your submission, please upload your figure files to the Preflight Analysis and Conversion Engine (PACE) digital diagnostic tool, https://pacev2.apexcovantage.com/. PACE helps ensure that figures meet PLOS requirements. To use PACE, you must first register as a user. Registration is free. Then, login and navigate to the UPLOAD tab, where you will find detailed instructions on how to use the tool. If you encounter any issues or have any questions when using PACE, please email PLOS at . PACE helps ensure that figures meet PLOS requirements. To use PACE, you must first register as a user. Registration is free. Then, login and navigate to the UPLOAD tab, where you will find detailed instructions on how to use the tool. If you encounter any issues or have any questions when using PACE, please email PLOS at . PACE helps ensure that figures meet PLOS requirements. To use PACE, you must first register as a user. Registration is free. Then, login and navigate to the UPLOAD tab, where you will find detailed instructions on how to use the tool. If you encounter any issues or have any questions when using PACE, please email PLOS at . PACE helps ensure that figures meet PLOS requirements. To use PACE, you must first register as a user. Registration is free. Then, login and navigate to the UPLOAD tab, where you will find detailed instructions on how to use the tool. If you encounter any issues or have any questions when using PACE, please email PLOS at figures@plos.org. Please note that Supporting Information files do not need this step.. Please note that Supporting Information files do not need this step.. Please note that Supporting Information files do not need this step.. Please note that Supporting Information files do not need this step.

# Author comment: Author response to Decision Letter 1

**DOI:** 10.1371/journal.pone.0345173.r002

29 Oct 2025

All comments were attended to and the full response submitted under the document titled "Response to Reviewers"

Attachment Submitted filename: Response to Reviewers.docx

# Peer review: Decision Letter 1

**DOI:** 10.1371/journal.pone.0345173.r003

**Authors:** Vazrick Nazari

14 Jan 2026

Thank you for submitting your manuscript to PLOS ONE. After careful consideration, we feel that it has merit but does not fully meet PLOS ONE’s publication criteria as it currently stands. Therefore, we invite you to submit a revised version of the manuscript that addresses the points raised during the review process.

Please submit your revised manuscript by Feb 28 2026 11:59PM. If you will need more time than this to complete your revisions, please reply to this message or contact the journal office at plosone@plos.org. When you're ready to submit your revision, log on to https://www.editorialmanager.com/pone/ and select the 'Submissions Needing Revision' folder to locate your manuscript file.. When you're ready to submit your revision, log on to https://www.editorialmanager.com/pone/ and select the 'Submissions Needing Revision' folder to locate your manuscript file.. When you're ready to submit your revision, log on to https://www.editorialmanager.com/pone/ and select the 'Submissions Needing Revision' folder to locate your manuscript file.. When you're ready to submit your revision, log on to https://www.editorialmanager.com/pone/ and select the 'Submissions Needing Revision' folder to locate your manuscript file.

- A letter that responds to each point raised by the academic editor and reviewer(s). You should upload this letter as a separate file labeled 'Response to Reviewers'.
- A marked-up copy of your manuscript that highlights changes made to the original version. You should upload this as a separate file labeled 'Revised Manuscript with Track Changes'.
- An unmarked version of your revised paper without tracked changes. You should upload this as a separate file labeled 'Manuscript'.

If you would like to make changes to your financial disclosure, please include your updated statement in your cover letter. Guidelines for resubmitting your figure files are available below the reviewer comments at the end of this letter.

If applicable, we recommend that you deposit your laboratory protocols in protocols.io to enhance the reproducibility of your results. Protocols.io assigns your protocol its own identifier (DOI) so that it can be cited independently in the future. For instructions see: https://journals.plos.org/plosone/s/submission-guidelines#loc-laboratory-protocols. Additionally, PLOS ONE offers an option for publishing peer-reviewed Lab Protocol articles, which describe protocols hosted on protocols.io. Read more information on sharing protocols at . Additionally, PLOS ONE offers an option for publishing peer-reviewed Lab Protocol articles, which describe protocols hosted on protocols.io. Read more information on sharing protocols at . Additionally, PLOS ONE offers an option for publishing peer-reviewed Lab Protocol articles, which describe protocols hosted on protocols.io. Read more information on sharing protocols at . Additionally, PLOS ONE offers an option for publishing peer-reviewed Lab Protocol articles, which describe protocols hosted on protocols.io. Read more information on sharing protocols at https://plos.org/protocols?utm_medium=editorial-email&utm_source=authorletters&utm_campaign=protocols....

We look forward to receiving your revised manuscript.

Kind regards,

Vazrick Nazari, PhD

Academic Editor

PLOS One

Journal Requirements:

1. If the reviewer comments include a recommendation to cite specific previously published works, please review and evaluate these publications to determine whether they are relevant and should be cited. There is no requirement to cite these works unless the editor has indicated otherwise.

[Note: HTML markup is below. Please do not edit.]

Reviewer's Responses to Questions

**Comments to the Author**

Reviewer #1: (No Response)

Reviewer #3: (No Response)

Reviewer #4: (No Response)

**********

2. Is the manuscript technically sound, and do the data support the conclusions?

Reviewer #1: Yes

Reviewer #3: Partly

Reviewer #4: Yes

**********

3. Has the statistical analysis been performed appropriately and rigorously?

Reviewer #1: Yes

Reviewer #3: N/A

Reviewer #4: Yes

**********

4. Have the authors made all data underlying the findings in their manuscript fully available?

The PLOS Data policy requires authors to make all data underlying the findings described in their manuscript fully available without restriction, with rare exception (please refer to the Data Availability Statement in the manuscript PDF file). The data should be provided as part of the manuscript or its supporting information, or deposited to a public repository. For example, in addition to summary statistics, the data points behind means, medians and variance measures should be available. If there are restrictions on publicly sharing data—e.g. participant privacy or use of data from a third party—those must be specified.requires authors to make all data underlying the findings described in their manuscript fully available without restriction, with rare exception (please refer to the Data Availability Statement in the manuscript PDF file). The data should be provided as part of the manuscript or its supporting information, or deposited to a public repository. For example, in addition to summary statistics, the data points behind means, medians and variance measures should be available. If there are restrictions on publicly sharing data—e.g. participant privacy or use of data from a third party—those must be specified.

Reviewer #1: Yes

Reviewer #3: No

Reviewer #4: (No Response)

**********

5. Is the manuscript presented in an intelligible fashion and written in standard English?

Reviewer #1: Yes

Reviewer #3: Yes

Reviewer #4: Yes

**********

Reviewer #1: (No Response)

Reviewer #3: The manuscript entitled “An overview of DNA barcoding of biodiversity in South Africa” provides a comprehensive synthesis of DNA barcoding efforts in South Africa by integrating a literature review with a large-scale analysis of public sequence repositories (BOLD and GenBank). The topic is timely and relevant, the datasets are extensive, and the study fits well within the scope of PLOS ONE. Overall, the work is technically sound and has the potential to make a valuable contribution to understanding progress and remaining gaps in DNA barcoding coverage in South Africa. However, several aspects of the methodology and interpretation require clarification or more cautious framing to improve transparency, reproducibility, and robustness of the conclusions.

Study design and terminology

The manuscript would benefit from a clearer statement of its study design, particularly in the abstract and introduction. It is not always clear whether the study should be interpreted primarily as a review, a data-driven gap analysis based on public sequence repositories, or a hybrid synthesis combining both approaches. Clarifying this would help readers better understand the scope and limitations of the work. In addition, some terminology is potentially ambiguous (e.g. “South African species barcode records,” “taxonomic representation”) and should be defined more precisely to avoid misinterpretation.

Checklist compilation and taxonomic standardisation

The checklist verification process draws on multiple authoritative sources and expert input, which is a major strength. However, checklist sources and compilation procedures differ substantially among plants, fungi, and animals. These differences should be more explicitly acknowledged and discussed, as they may affect comparability across taxonomic groups in subsequent gap analyses. The decision to treat taxa with qualifiers such as cf., aff., or nov. as valid species names, as well as the collapsing of infraspecific taxa to species level, is unconventional and may inflate estimates of species-level barcode coverage. These choices require clearer justification and a more explicit discussion of their implications for interpreting taxonomic gaps. Similarly, the handling of alien species across multiple databases should be described in greater detail, particularly where classifications may conflict among sources.

BOLD and GenBank data retrieval

The procedures used to mine data from BOLD and GenBank are generally appropriate, but additional detail is needed to ensure reproducibility. In particular, reliance on the search term “South Africa” in geography or metadata fields may exclude records with incomplete or inconsistently formatted locality information. The authors should clarify which metadata fields were queried and whether alternative filters (e.g. country codes or coordinate-based selection) were considered. The criteria for retaining or excluding specific data fields, as well as any quality control steps applied during data cleaning and standardisation, should also be described more explicitly. The comparison between total GenBank records and those mined by BOLD is central to the study’s conclusions; therefore, the method used to quantify discrepancies should be explained in more detail, including whether differences may stem from non-barcode gene regions or insufficient metadata rather than true data gaps.

Geographic analyses and institutional contributions

The geographic analysis provides useful insights but relies on several assumptions that should be more clearly justified. Assigning centroid coordinates to records lacking GPS data may bias provincial patterns, particularly for large or environmentally heterogeneous provinces. The authors should justify this approach and discuss its limitations, or clarify whether analyses were repeated excluding centroid-based records. Restricting geographic analyses to BIN-assigned records may also bias results against taxa such as plants and fungi, and this limitation should be explicitly acknowledged. In addition, institutional and geographic contributions inferred from the institution_storing field require clearer explanation, as storage location does not always reflect sampling locality or data ownership.

Interpretation of BIN-based metrics

Several key conclusions rely on the assumption that each Barcode Index Number (BIN) represents a distinct species lineage. While BINs are a useful proxy, this relationship is taxon-dependent and subject to methodological thresholds. Estimates of barcoding completeness based on BIN counts should therefore be presented more cautiously as approximations rather than direct measures of species-level coverage. Similarly, BIN splits and merges should not be interpreted solely as evidence of taxonomic deficiencies, as they may also reflect biological processes such as recent divergence or introgression.

Data availability

The manuscript makes substantial use of publicly available data and provides supporting tables and files. However, a large proportion of BOLD records referenced in the analyses remain private and cannot be independently accessed. While this limitation is acknowledged, it should be more clearly addressed in the context of the PLOS data availability policy, and the authors should clarify which conclusions rely on inaccessible data versus fully public datasets.

Language and presentation

The manuscript is generally well written and intelligible, with standard scientific English throughout. Minor wording issues and some long or complex sentences could be improved for clarity during revision, but no major language problems were identified.

Conclusion

In summary, this manuscript addresses an important topic and is based on substantial datasets. With clearer methodological descriptions, more explicit discussion of assumptions and limitations, and more cautious interpretation of BIN-based metrics, the study would be substantially strengthened and well suited for publication in PLOS ONE.

Reviewer #4: (No Response)

**********

what does this mean?). If published, this will include your full peer review and any attached files.). If published, this will include your full peer review and any attached files.). If published, this will include your full peer review and any attached files.). If published, this will include your full peer review and any attached files.

If you choose “no”, your identity will remain anonymous but your review may still be made public.

**Do you want your identity to be public for this peer review?** For information about this choice, including consent withdrawal, please see our For information about this choice, including consent withdrawal, please see our For information about this choice, including consent withdrawal, please see our For information about this choice, including consent withdrawal, please see our Privacy Policy..

Reviewer #1: No

Reviewer #3: No

Reviewer #4: No

**********

[NOTE: If reviewer comments were submitted as an attachment file, they will be attached to this email and accessible via the submission site. Please log into your account, locate the manuscript record, and check for the action link "View Attachments". If this link does not appear, there are no attachment files.]

To ensure your figures meet our technical requirements, please review our figure guidelines: https://journals.plos.org/plosone/s/figures

You may also use PLOS’s free figure tool, NAAS, to help you prepare publication quality figures: https://journals.plos.org/plosone/s/figures#loc-tools-for-figure-preparation.

NAAS will assess whether your figures meet our technical requirements by comparing each figure against our figure specifications.

Attachment Submitted filename: 20250111_PLODONE_review.docx

Attachment Submitted filename: Comments to the authors_Plos One.pdf

# Author comment: Author response to Decision Letter 2

**DOI:** 10.1371/journal.pone.0345173.r004

28 Feb 2026

We have provided a detailed response in the "Response to Reviewer" document which is attached. We really appreciate the much focused and specific comments to the manuscript.

Attachment Submitted filename: Response_to_Reviewers_auresp_2.docx

# Editor report: Decision Letter 2

**DOI:** 10.1371/journal.pone.0345173.r005

**Authors:** Vazrick Nazari

3 Mar 2026

An overview of DNA barcoding of biodiversity in South Africa

PONE-D-25-08952R2

Dear Dr. Kgatla,

We’re pleased to inform you that your manuscript has been judged scientifically suitable for publication and will be formally accepted for publication once it meets all outstanding technical requirements.

Within one week, you’ll receive an e-mail detailing the required amendments. When these have been addressed, you’ll receive a formal acceptance letter and your manuscript will be scheduled for publication.

An invoice will be generated when your article is formally accepted. Please note, if your institution has a publishing partnership with PLOS and your article meets the relevant criteria, all or part of your publication costs will be covered. Please make sure your user information is up-to-date by logging into Editorial Manager at Editorial Manager® and clicking the ‘Update My Information' link at the top of the page. For questions related to billing, please contact  and clicking the ‘Update My Information' link at the top of the page. For questions related to billing, please contact  and clicking the ‘Update My Information' link at the top of the page. For questions related to billing, please contact  and clicking the ‘Update My Information' link at the top of the page. For questions related to billing, please contact billing support....

If your institution or institutions have a press office, please notify them about your upcoming paper to help maximize its impact. If they’ll be preparing press materials, please inform our press team as soon as possible -- no later than 48 hours after receiving the formal acceptance. Your manuscript will remain under strict press embargo until 2 pm Eastern Time on the date of publication. For more information, please contact onepress@plos.org.

Kind regards,

Vazrick Nazari, PhD

Academic Editor

PLOS One

Additional Editor Comments (optional):

Reviewers' comments:

# Editor report: Acceptance letter

**DOI:** 10.1371/journal.pone.0345173.r006

**Authors:** Vazrick Nazari

PONE-D-25-08952R2

PLOS One

Dear Dr. Kgatla,

I'm pleased to inform you that your manuscript has been deemed suitable for publication in PLOS One. Congratulations! Your manuscript is now being handed over to our production team.

At this stage, our production department will prepare your paper for publication. This includes ensuring the following:

* All references, tables, and figures are properly cited

* All relevant supporting information is included in the manuscript submission,

* There are no issues that prevent the paper from being properly typeset

You will receive further instructions from the production team, including instructions on how to review your proof when it is ready. Please keep in mind that we are working through a large volume of accepted articles, so please give us a few days to review your paper and let you know the next and final steps.

Lastly, if your institution or institutions have a press office, please let them know about your upcoming paper now to help maximize its impact. If they'll be preparing press materials, please inform our press team within the next 48 hours. Your manuscript will remain under strict press embargo until 2 pm Eastern Time on the date of publication. For more information, please contact onepress@plos.org.

You will receive an invoice from PLOS for your publication fee after your manuscript has reached the completed accept phase. If you receive an email requesting payment before acceptance or for any other service, this may be a phishing scheme. Learn how to identify phishing emails and protect your accounts at https://explore.plos.org/phishing.

If we can help with anything else, please email us at customercare@plos.org.

Thank you for submitting your work to PLOS ONE and supporting open access.

Kind regards,

PLOS ONE Editorial Office Staff

on behalf of

Dr. Vazrick Nazari

Academic Editor

PLOS One
